# Musashi-1 promotes stress-induced tumor progression through recruitment of AGO2

**DOI:** 10.7150/thno.35895

**Published:** 2020-01-01

**Authors:** Hsiao-Yun Chen, Mong-Lien Wang, Benoit Laurent, Chih-Hung Hsu, Ming-Teh Chen, Liang-Ting Lin, Jia Shen, Wei-Chao Chang, Jennifer Hsu, Mien-Chie Hung, Yi-Wei Chen, Pin-I Huang, Yi-Ping Yang, Chung-Pin Li, Hsin-I Ma, Chung-Hsuan Chen, Wen-Chang Lin, Shih-Hwa Chiou

**Affiliations:** 1 Division of Basic Research, Department of Medical Research, Taipei Veterans General Hospital, Taipei, Taiwan.; 2 Institute of Clinical Medicine, School of Medicine, National Yang-Ming University, Taipei, Taiwan.; 3 Institute of Pharmacology, School of Medicine, National Yang-Ming University, Taipei, Taiwan.; 4 Institute of Food Safety and Health Risk Assessment, School of Pharmaceutical Sciences, National Yang-Ming University, Taipei, Taiwan.; 5 Boston Children Hospital and Harvard Medical School, Boston MA, USA.; 6 Department of Public Health, and Women's Hospital, Zhejiang University School of Medicine, Hangzhou, Zhejiang, China.; 7 Division of Newborn Medicine and Epigenetics Program, Department of Medicine, Boston Children's Hospital, Boston, and Department of Cell Biology, Harvard Medical School, Boston, Massachusetts, USA.; 8 Department of Neurosurgery, Taipei Veterans General Hospital, Taipei, Taiwan.; 9 Department of Molecular and Cellular Oncology, University of Texas MD Anderson Cancer Center, Houston, Texas, USA.; 10 Graduate Institute of Biomedical Sciences and Center for Molecular Medicine, China Medical University, Taichung, Taiwan.; 11 Cancer Center, Taipei Veterans General Hospital, Taipei, Taiwan.; 12 Department of Neurological Surgery, Tri-Service General Hospital and National Defense Medical Center, Taipei, Taiwan.; 13 Genomic Research Center, Academia Sinica, Taipei, Taiwan.; 14 Institute of Biomedical Sciences, Academia Sinica, Taipei, Taiwan.

**Keywords:** Musashi-1, Argonaute 2, RNA regulation, subcellular translocation, cancer, tumor recurrence

## Abstract

Carcinomatous progression and recurrence are the main therapeutic challenges frequently faced by patients with refractory tumors. However, the underlined molecular mechanism remains obscure.

**Methods**: We found Musashi-1 (MSI1) transported into cytosol under stress condition by confocal microscopy and cell fractionation. Argonaute 2 (AGO2) was then identified as a cytosolic binding partner of MSI1 by Mass Spectrametry, immunoprecipitation, and recombinant protein pull-down assay. We used RNA-IP to determine the MSI1/AGO2 associated regions on downstream target mRNAs. Finally, we overexpressed C-terminus of MSI1 to disrupt endogenous MSI1/AGO2 interaction and confirm it effects on tmor progression.

**Results**: Malignant tumors exhibit elevated level of cytosolic Musashi-1 (MSI1), which translocates into cytosol in response to stress and promote tumor progression. Cytosolic MSI1 forms a complex with AGO2 and stabilize or destabilize its target mRNAs by respectively binding to their 3´ untranslated region or coding domain sequence. Both MSI1 translocation and MSI1/AGO2 binding are essential for promoting tumor progression. Blocking MSI1 shuttling by either chemical inhibition or point mutation attenuates the growth of GBM-xenografts in mice. Importantly, overexpression of the C-terminus of MSI1 disrupts endogenous MSI1/AGO2 interaction and effectively reduces stress-induced tumor progression.

**Conclusion**: Our findings highlight novel molecular functions of MSI1 during stress-induced carcinomatous recurrence, and suggest a new therapeutic strategy for refractory malignancies by targeting MSI1 translocation and its interaction with AGOs.

## Introduction

RNA binding proteins (RBPs) play crucial roles in various cellular processes by regulating the post-transcriptional control of their mRNA targets, such as microRNA biogenesis, RNA localization, translation and stability 1-6. The RBP family of Musashi proteins, composed of Musashi-1 (MSI1) and Musashi-2, exerts an essential control over multiple cellular functions 7, such as the maintenance of self-renewal and pluripotency state in stem cells 8. Dysfunctions in the expression or activity of this family have been shown to lead to tumorigenesis of glioblastoma (GBM) or pancreatic ductal adenocarcinoma (PDAC) 9, 10. MSI1 was recently reported to directly target the 3' untranslated region (3' UTR) of its target mRNAs to suppress their translation 11. MSI1 also cooperates with LIN28 RBP to inhibit the post-transcriptional biogenesis of miRNAs in embryonic stem cells 12. Increasing evidence points to the role of MSI1 in tumorigenesis and cancer proliferation 13. High level of MSI1 expression has been observed in several tumor tissues 9, 10, 14-17, and is associated with poor survival of grade III/IV gliomas patients 18. Although these studies suggest the involvement of MSI1 in malignancy, its functional roles and molecular mechanisms underlying carcinomatous recurrence remain largely unknown.

The Argonaute (AGO) proteins, also part of the RBP family, play a central role in RNA silencing processes by mediating the decay and translational inhibition of their targets 19-21. In many carcinomas, AGO2 is found to be ectopically overexpressed 19, and several studies indicated that AGO2 could directly be involved in cancers progression by interacting with oncogenic factors like EGFR 22. AGO2 also responds to stress stimulation by remodeling its interactions with target mRNAs and by modulating their post-transcriptional control 23. By remodeling its occupancy on the 3´ UTR and coding sequence (CDS) region of target mRNAs, AGO2 adjusts the translation rate of specific group of genes 23. However, the mechanisms by which AGO2 coordinates the translation rate of specific targets in response to stresses in malignant progression are still unclear.

In this study, we report that, in response to stress, MSI1 translocates into the cytosol where it recruits AGO2 and post-transcriptionally regulates the expression of specific target mRNAs. The binding of MSI1/AGO2 to the 3´ UTR of target mRNAs enhances their degradation whereas binding to CDS prevents their rapid degradation. By coordinating the two mechanisms, MSI1/AGO2 complex enhances tumor proliferation and ensures cancer cell survival under hypoxia or chemodrug treatment. We also show that disrupting MSI1/AGO2 interaction by overexpressing C-terminal region of MSI1 decreases stress-induced tumorigenicity. Notably, overexpression of MSI1 C-terminus increased sensitivity to chemotherapeutic drugs, thus hindering the malignant progression of GBM, which opens the possibility to treat tumor recurrence via tackling the MSI1/AGO2 complex formation.

## Results

### Cytosolic translocation of MSI1 is essential for its pro-oncogenic effects under stress

**Figure 1:**
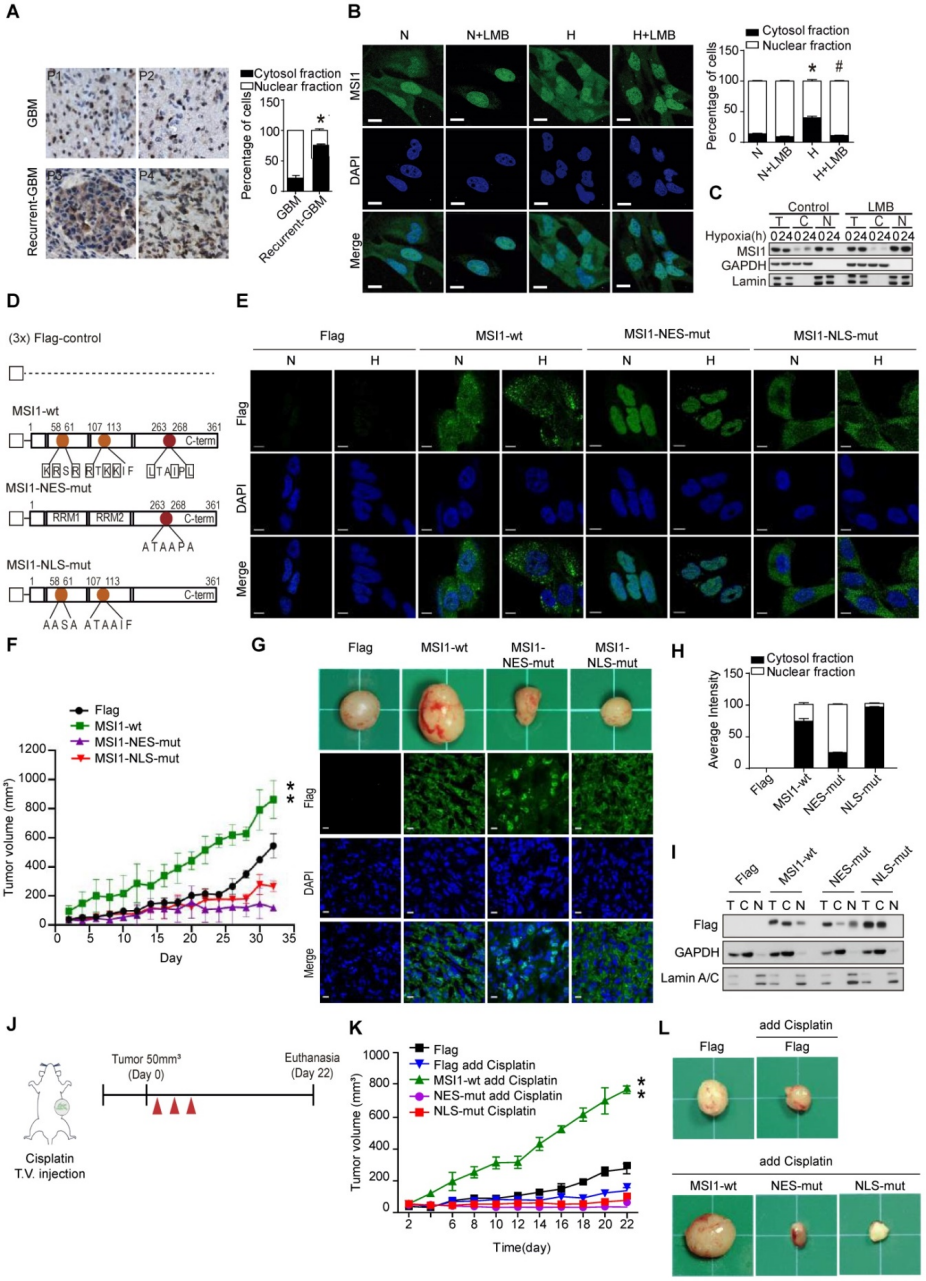
** Translocation of MSI1 into the cytosol correlates with tumor progression and cell proliferation under stress conditions. (A)** IHC staining for MSI1 in primary (n = 67) and recurrent (n = 32) GBM. Magnifying power: 200× (top) and 600× (bottom). Quantitation of cells expressing cytoplasmic or nuclear MSI1 is shown in the bar graph on the right. **(B)** 05MG cells pre-treated with or without nuclear export inhibitor leptomycinB (LMB) (10 ng/mL, 2 hr) under normoxia or hypoxia conditions for 24 hr were subjected to anti-MSI1 (green) immnunostaining and DAPI (blue) nuclear counterstaining. Images were acquired from Carl Zeiss confocal microscope system. The intensity of green fluorescence in nuclear and cytosol was quantified and shown as relative percentage in the graph at the right. **(C)** Total protein (T), nuclear (N), and cytoplasmic (C) fractionations of 05MG cells under normoxia or hypoxia (24 hr) in the presence or absence of LMB (10 ng/mL) were subjected to immunoblotting with MSI1, Lamin A/C (nuclear internal control) and GAPDH (cytosolic control) antibodies. **(D)** A schematic presentation showing the mutation sites in the NLS (orange) and NES (red) motifs of human MSI1. All constructs were sub-cloned into p-3xFlag-Myc-CMV expression vector. **(E)** 05MG cells stably transfected with the Flag-control, Flag-tagged MSI1-wt, MSI1-NES-mut, or MSI1-NLS-mut were subjected to normoxia (N) or hypoxia **(H)** treatment for 24 hr and then immunostained with anti-Flag antibody (green). Images were acquired from Carl Zeiss confocal microscope system, and the quantification of fluorescent intensity in the nuclear and cytosolic compartments was shown as relative percentage in the graph at the left. **(F)** Null mice were subcutaneously transplanted with 05MG/Flag-control, 05MG/MSI1-wt, 05MG/MSI1-NES-mut or 05MG/MSI1-NLS-mut cells. Tumor size was measure with a caliper at the indicated time points. The 05MG/MSI1-NES-mut and 05MG/MSI1-NLS-mut tumors showed similar growth curves as the 05MG/Flag-control cells, while the 05MG/MSI1-wt tumor grew much more rapidly. N = 6. **P < 0.05 vs. 05MG/Flag-controlcells. **(G)** Xenograft tumors were excised (top), and tumor tissues were subjected to immunostaining to evaluate the expression and distribution of Flag-control, Flag-tagged MSI1-wt, MSI1-NES-mut, and MSI1-NLS-mut proteins (bottom). Images were acquired from Carl Zeiss confocal microscope system. **(H)** The intensity of green fluorescence in nuclear and cytosol was quantified and shown as relative percentage in the graph at the right. **(I)** Tumor tissue were harvested and homogenized. Whole-tumor lysates were subjected to Western blot analysis. Data represent the mean ± S.D. of three independent experiments performed in triplicate. **(J)** A schematic depicting the experimental design for subcutaneously transplanted. **(K-L)** Null mice were subcutaneously transplanted with 05MG/Flag-control, 05MG/MSI1-wt, 05MG/MSI1-NES-mut, or 05MG/MSI1-NLS-mut cells. Two days after the tumor size reached 50 mm^3^, mice started cisplatin (20 mg/kg) or PBS administered via tail-vein injection for total 3 times with 2-day interval. The tumor size was measured with a caliper at the indicated time points. Xenograft tumors were excised 40 days after DDP treatment. N = 6, **P < 0.05.

Overexpression of MSI1 has been reported in several tumor tissues 9, 10, 13-17. Our previous studies reported the correlation between MSI1 expression and GBM migration, drug resistance, tumor progression; but little is known about the underlying mechanism 24-26. We first examined the correlation between MS1I and tumor progression by immunohistochemical (IHC) staining on a small cohort of glioma patient samples. We found that high levels of MSI1 expression positively correlated with grade of primary CNS malignancy (lower in 12 meningioma and 14 low grade glioma but highly expressed in 33 grade 4 GBM; Figure S1A-B). Further, we overexpressed or depleted MSI1 in 05MG cell lines to determine the proliferation, anti-apoptosis and tumor progression ability. Here, overexpression of MSI1 increased the colony number, anti-apoptosis percentage and tumor volume then Flag-control (Figure S1C-F), whereas depletion of MSI1 showed opposite results (Figure S1G-J). Intriguingly, we also observed a significant proportion of MSI1 proteins in the cytosol in recurrent glioma samples compared with the non-recurrent samples (Figure 1A). We asked whether MSI1 could be dynamically regulated in response to hypoxic or chemotherapeutic agents. To address this possibility, we first exposed 05MG cells, human glial cells derived from a patient with glioblastoma (GBM), to hypoxic treatment. Cell exposure to hypoxia did not affect the total level of MSI1, but increased MSI1 levels in the cytosolic compartment (Figure 1B-C). Similar results were obtained with primary GBM cells (Figure S2A-B) and pancreatic ductal adenocarcinoma (PDAC) cell lines (Figure S2C-D). Addition of Leptomycin B (LMB), an inhibitor of the nuclear export receptor CRM1, strongly reduced MSI1 translocation into the cytosol upon hypoxic and cisplatin treatment (Figure 1B-C and Figure S2E), suggesting an active and CRM1-dependent MSI1 translocation in response to environmental stress.

Subcellular localization is generally relied on a nuclear localization signal (NLS) and a nuclear export signal (NES). Two NLS sites have been reported in the N-terminal domain of MSI1 27 (Figure 1D). We identified a potential NES motif (263-LTAIPL-268) within the C-terminus of MSI1 and confirmed the functionality of this motif (Figure 1D and Figure S2F). Mutations in the NLS and NES motifs of MSI1 were generated; Flag-control, Flag-tagged wild-type MSI1 (MSI1-wt), NES-mutant MSI1 (MSI1-NES-mut) and NLS-mutant MSI1 (MSI1-NLS-mut) were stably expressed in GBM cells (Figure 1E). First we confirmed no differences on the protein and RNA expression levels between these stable clones (Figure S3 A-B). Further, under hypoxic conditions, MSI1-NES-mut and MSI1-NLS-mut remained respectively located in the nucleus and cytosol while MSI1-wt translocated into the cytosol (Figure 1E and Figure S3C), suggesting that the NES motif plays an active role in the subcellular translocation of MSI1 upon stress treatment.

We next investigated the biological consequences of MSI1 translocation. In vitro functional assays showed that cells overexpressing MSI1-wt exhibited decreased apoptosis, and increased proliferation and viability under hypoxia compared with Flag-control, MSI1-NES-mut, MSI1-NLS-mut overexpressing cells and MSI1-depleted cells (Figure S 3D-F). Consistently, in vivo studies revealed that xenografts of GBM cells overexpressing MSI1-wt grew significantly bigger tumors than that of Flag-control or MSI1-mutant GBM cells (Figure 1F-G). Subcellular localization of MSI1 and its mutants in xenografts (Figure 1G-I) was consistent with that observed in Figure 1e. We further explored the consequences of MSI1 shuttling in GBM under oxidative stress by treating subcutaneous xenografts with cisplatin (Figure 1J). Compared with our previous experiments (Figure 1F-G), cisplatin treatment enhanced tumor growth of xenografts overexpressing MSI1-wt (774.365 mm^3^ *vs* 477.437 mm^3^ tumor volume at day 22) (Figure 1K-I). Mice intracranially implanted with xenografts overexpressing MSI1-wt and sequentially treated with cisplatin showed an outrageous tumor invasion compared with other groups (Figure 1K-I), suggesting that the nuclear-cytoplasmic shuttling of MSI1 strictly governs its biological function in tumorigenicity. Together, our findings showed that stress-induced translocation of MSI1 is required for its pro-oncogenic functions.

### Cytosolic MSI1 directly binds AGO2 and its target mRNAs under stress condition

**Figure 2:**
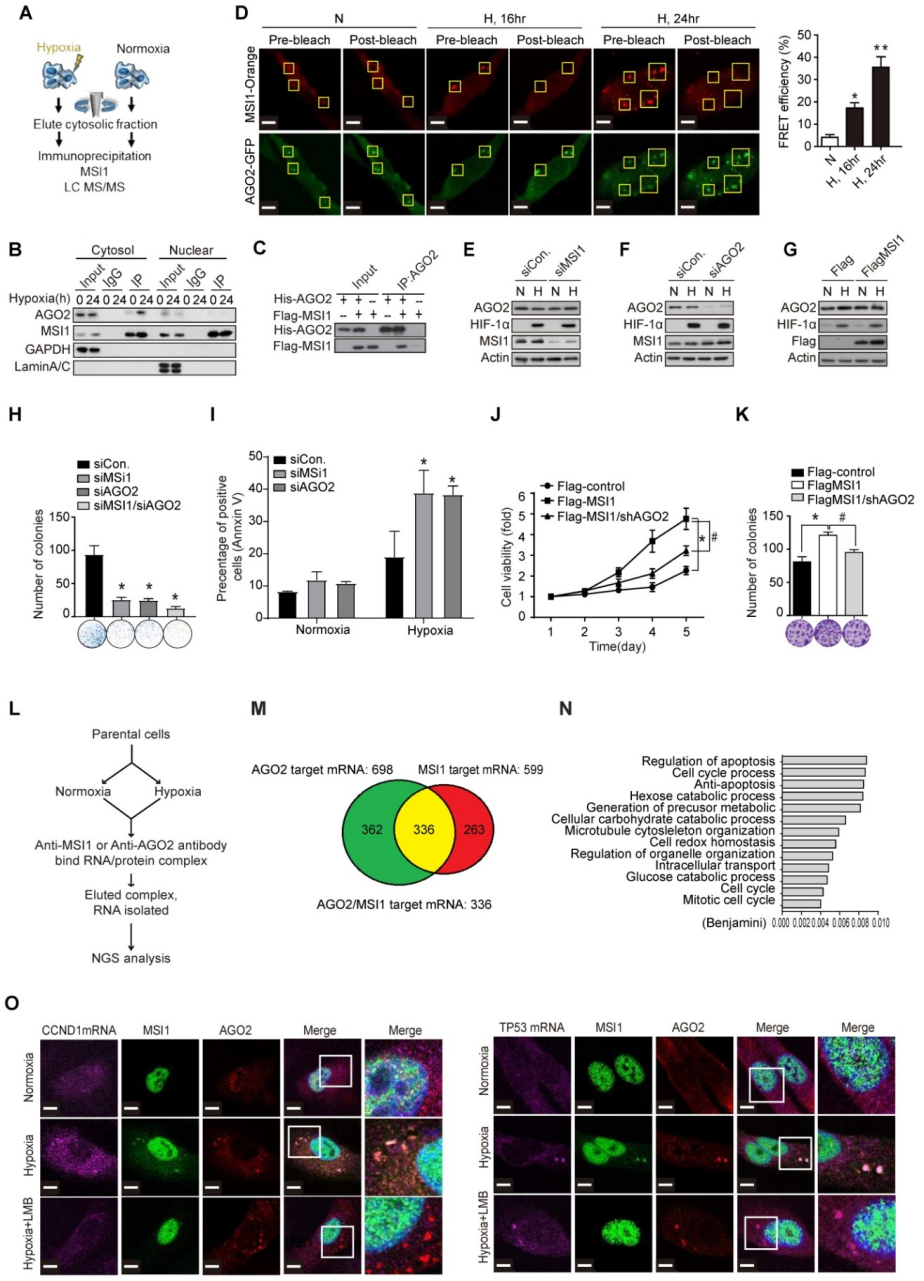
** MSI1 interacts with AGO2 and binds to their common downstream mRNA targets under hypoxia. (A)** A schematic illustrating the procedure for identifying hypoxia-induced binding partners of MSI1. **(B)** Co-immunoprecipitation of endogenous AGO2 with MSI1 antibody in the cytosol or nuclear fraction of 05MG cells under hypoxia for indicated period of time. **(C)** In vitro binding assay of purified baculovirus-expressed His-tagged AGO2 and Flag-tagged MSI1 proteins. **(D)** 05MG cells-expressing FRET pairs of MSI1-orange and AGO2-GFP were bleached at the region of interest (ROI) indicated by yellow boxes. Unbleached controls (pre-bleach) were also shown in parallel. Left, representative images of MSI1 (orange) and AGO2 (green) before and after photobleaching experiments. Right, quantification of FRET photobleaching experiments was performed by calculating FRET efficiencies for the FRET pairs MSI1 (orange)-AGO2 (green). **(E-G)** Western blotting confirmed endogenous expression levels of MSI1, AGO2, HIF-1α and actin in MSI1-knockdown cells **(E)**, AGO2-knockdown cells **(F),** and MSI1-overexpressed cells **(G). (H)** 05MG with AGO2-knockdown were subjected to an MTT viability assay. The relative fold change of the number of viable cells in each day was presented in the graph. **(I)** The percentage of apoptotic cells of control, MSI1-knockdown and AGO2-knockdown cells were determined by external Annexin-V under normoxiac and hypoxic conditions. **(J)** 05MG/Flag-control, 05MG/Flat-MSI1-wt, and 05MG/MSI1-wt with AGO2-knockdown (Flag-MSI1/shAGO2) were subjected to an MTT viability assay. The relative fold change of the numbers of viable cells in each day was presented in the graph. **(K)** 05MG/Flag-control, 05MG/MSI1-wt and 05MG/MSI1-wt/shAGO2 cells were subjected to colony formation assay for 10 days, and the numbers of colony were quantitated by ImageJ software. **(L)** Flow-chart of preparing RNA-binding protein immunoprecipitation (RIP) samples for NGS analysis (RIP-seq). **(M)** Intersection of mRNA targeted by MSI1 and AGO2. **(N)** Gene ontology (GO) enrichment analysis was conducted by DAVID software according to the category of biological processes. Benjamini ≤ 0.05 is selected as interesting GO. The GO accession, name, and the corresponding p-value were shown in the graph. **(O)** 05MG cells pre-treated with or without LBM (10 ng/mL, 2 hours) and cultured in hypoxia condition for 24 hours were stained for TP53 and CCND1 mRNAs (TAS-cy5, cherry-red), MSI1 (green), and AGO2 (red). Merged images of co-localization of MSI1/AGO2/mRNA (white) by confocal microscopy are shown.

To address the underlying molecular mechanisms by which MSI1 shuttling promotes stress-induced tumor progression, we characterized MSI1 interacting proteins by mass spectrometry analysis. The Flag-tagged MSI1 protein complex in the cytosolic fraction of 05MG cells under normoxia or hypoxia was purified and characterized (Figure 2A and Figure S4A). We found 142 proteins potentially associate with MSI1 in the cytosol. We were particularly interested in those proteins that are related to stress response, such as eIF3A, PABP, PKR, GCN2, and AGO2 (Figure S4B). In our immunoprecipitation data, we found that the interactions of MSI1 and AGO2 sustained RNase A treatment, suggesting an RNA independent manner of this RNA binding proteins interaction (Figure S4C). Moreover, we found that hypoxic stress significantly enhanced the recruitment of AGO2 to cytosolic MSI1 in GBM and PDAC cancer cells (Figure 2B and Figure S4D-E). In vitro binding assay confirmed the direct interaction between recombinant MSI1 and AGO2 (Figure 2C). Fluorescence Resonance Energy Transfer (FRET) microscopy (Figure 2D and Figure S4F), and confocal microscopy in cisplatin-treated (Figure S4G) and hypoxia-treated (Figure S4H) cells confirmed the stress-induced interaction between MSI1 and AGO2.

We next investigated whether AGO2 is essential for the oncogenic functions of MSI1. First we showed overexpression of Flag-control, MSI1-wt, MSI1-NES-mut, or MSI1-NLS-mut did not change the protein and RNA levels of AGO1 and AGO2 (Figure S5A-C). Knockdown and overexpression of MSI1 did not affect the protein and RNA levels of AGO2, neither did knockdown of AGO2 affect the expression of MSI1 (Figure 2 E-G). However, knockdown of MSI1 or AGO2 suppressed cell viability and enhanced apoptosis (Figure 2H-I). We also showed that AGO2 knockdown (Figure S5D) in MSI1-overexpressed cells suppressed the viability (Figure 2J) and proliferation (Figure 2K) through enhanced apoptosis (Figure S5E). Concomitantly, in vivo studies showed that AGO2 knockdown abolished the MSI1-enhanced tumor growth (Figure S5C). These data showed that AGO2 is an important downstream effector of MSI1 involved in cancer development.

To characterize the functional roles of stress-induced AGO2-MSI1 interaction, we performed RNA-binding protein immunoprecipitation sequencing (RIP-Seq) by pull-down MSI1 and AGO2 respectively, and identified 336 common mRNA targets bound by MSI1/AGO2 complex (Figure 2L-M). Gene Ontology (GO) analysis of the mRNAs associated with both MSI1 and AGO2 showed enrichment in cell cycle progression and apoptosis pathways (Figure 2N), consistent with the cellular phenotype we observed. We further confirmed the binding of MSI1/AGO2 complex to some of the mRNA targets upon hypoxia (Figure 2O and Figure S6A). Conclusively, under stress condition like hypoxia, MSI1 forms a complex with AGO2 and binds to its target mRNA.

### The MSI1/AGO2 complex regulates the stability of its target mRNA through CDS or 3'UTR binding

**Figure 3:**
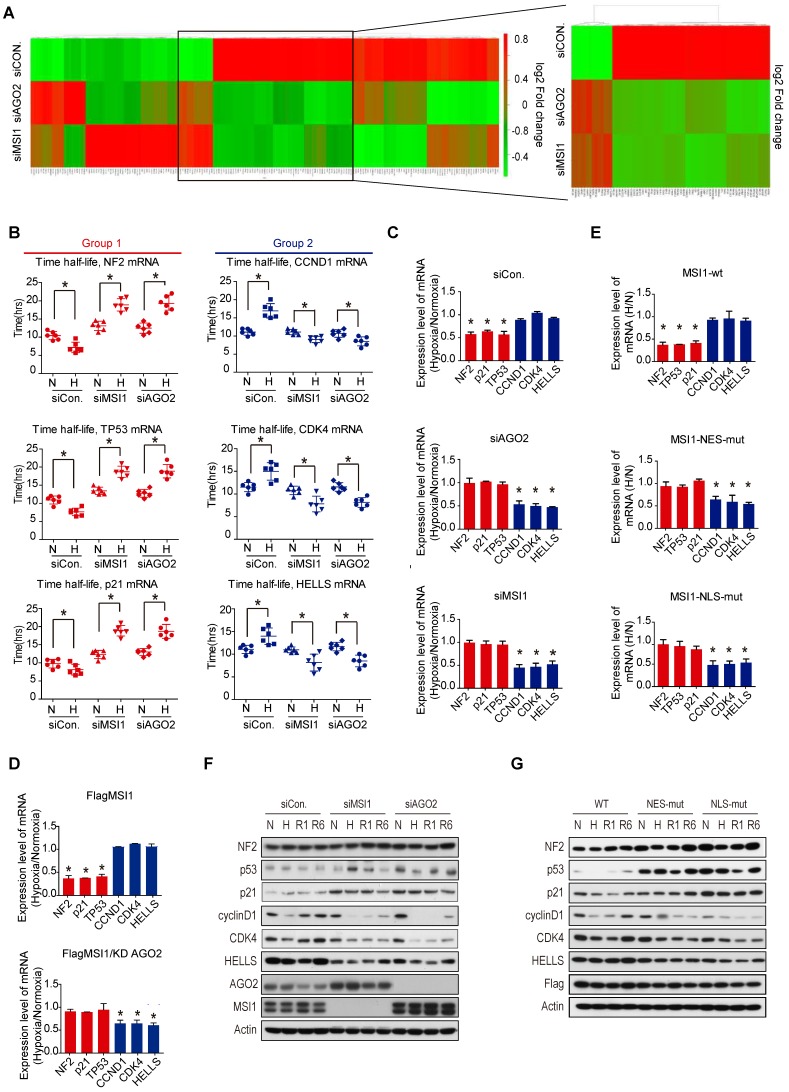
** MSI1/AGO2 mediate the stability of downstream mRNA targets. (A)** Parental and MSI1- or AGO2-knockdown cells under normoxia and hypoxia conditions were subjected to a gene expression microarray. Bioinformatics analysis of the microarray data with focus on the 336 common targets of MSI1 and AGO2 identified by RIP-Seq showed the hierarchical clustering these common targets in the heat map. The red and green colors respectively indicate the differentially up or downregulated genes. Each group were done in three distinct biological replicates and the means signals were transformed to the log2 scale. **(B)** Actinomycin D (Act. D, 5 μg/ml) was added to parental and MSI1- or AGO2-knockdown cells for the indicated times. We compared the half-life distribution of TP53, NF2, CDKN1A, CCND1, CDK4 and HELLS mRNA levels between parental and MSI1- or AGO2-knockdown cells. The RNA expression levels are shown below each the respective box-plots. **(C, E)** Parential and MSI1- or AGO2-knockdown 05MG cells, as well as MSI1-wt, MSI1-NES-mut, and MSI1-NLS-mut transfected 05MG cells were treated with normoxia or hypoxia conditions for 24 hours. Purified RNA was subjected to RT-PCR with primers specific to TP53, NF2, CDKN1A, CCND1, CDK4 and HELLS. The mRNA levels under hypoxia were normalized by that under normoxia and shown as relative value in the chart. **(D)** 05MG/MSI1-wt and 05MG/MSI1-wt/shAGO2 cells were treated with normoxia or hypoxia conditions for 24 hours. Purified RNA was subjected to quantitative RT-PCR with primers specific to TP53, NF2, CDKN1A, CCND1, CDK4 and HELLS. The mRNA levels under hypoxia were normalized by that under normoxia and shown as relative value in the chart. * P<0.05. **(F)** 05MG with AGO2- or MSI1-knockdown, and **(G)** MSI1-wt, MSI1-NES-mut, or MSI1-NLS-mut overexpression under normoxia or hypoxia for 24 hours, or recovery from hypoxia condition for 1 and 6 hours, were subjected to Western blotting with antibody of NF2, p53, p21, cyclin D1, CDK4, HELLS, AGO2, MSI1, Flag and actin.

We then investigated the impact of MSI1/AGO2 binding on the expression level of the 336 common target mRNAs. Steady mRNA levels were profiled by microarrays in control, MSI1- and AGO2-knockdown cells cultured under normoxia or hypoxia. Intriguingly, we observed two distinct groups of mRNA targets, with the first group (group 1) enriched in apoptotic genes exhibiting increased mRNA level after knockdown of MSI1 and AGO2 under hypoxia, and the second group (group 2) of genes mainly involved in cell cycle regulation showing opposite regulatory trend (Figure 3A). We selected three mRNA targets from each group - *NF2*, *TP53*, and *p21* from group 1, and* CCND1*, *CDK4*, *HELLS* from group 2 - and evaluated their degradation rate under normoxia and hypoxia by treating cells with actinomycin D (Figure 3B). In control cells, hypoxic stress decreased the half-life of group1 mRNAs while increased the stability of group2 mRNAs. In contrast, we observed the opposite effect in MSI1- and AGO2-knockdown cells (Figure 3B), suggesting that MSI1/AGO2 complex could stabilize in response to stress a subset of mRNA targets related to cell cycle (group 1) to subsequently promote tumor progression. Along with this idea, MSI1/AGO2 binding could also negatively regulate the stability of another subset of mRNA targets related to apoptosis (group 2) to ensure cancer cell survival. We verified our hypothesis by qPCR and confirmed the existence of two distinct types of regulation: 1) the stability of mRNA targets from group 1 decreased in response to hypoxia and 2) the mRNA targets from group 2 remained expressed at similar levels after hypoxia (Figure 3C). Of note, AGO2 is essential for the stress-induced and MSI1-mediated regulation of downstream mRNAs as AGO2-knockdown in MSI1-overexpressed cells abrogated the regulation of group1 and group 2 mRNA stability (Figure 3D). Consistent with cytosolic MSI1-AGO2 interaction, we found that cells overexpressing MSI1 mutants, with detoured subcellular localization, recaptured the functional consequence on mRNA stability in a similar manor to the one caused by knockdown of MSI1 in response to hypoxia (Figure 3E). Finally, we also determined the protein expression level of group 1 and group 2. Intriguingly, we noticed that the group1 targets of *NF2*, *TP53*, and *p21* were increased while the group 2 targets of *CCND1*, *CDK4* and *HELLS* were decreased in MSI1-depleted or AGO2-depleted cells under hypoxia and recovery condition (Figure 3F). The same results were also observed in the MSI1-NES-mut and MSI1-NLS-mut transfected cells (Figure 3G).

**Figure 4:**
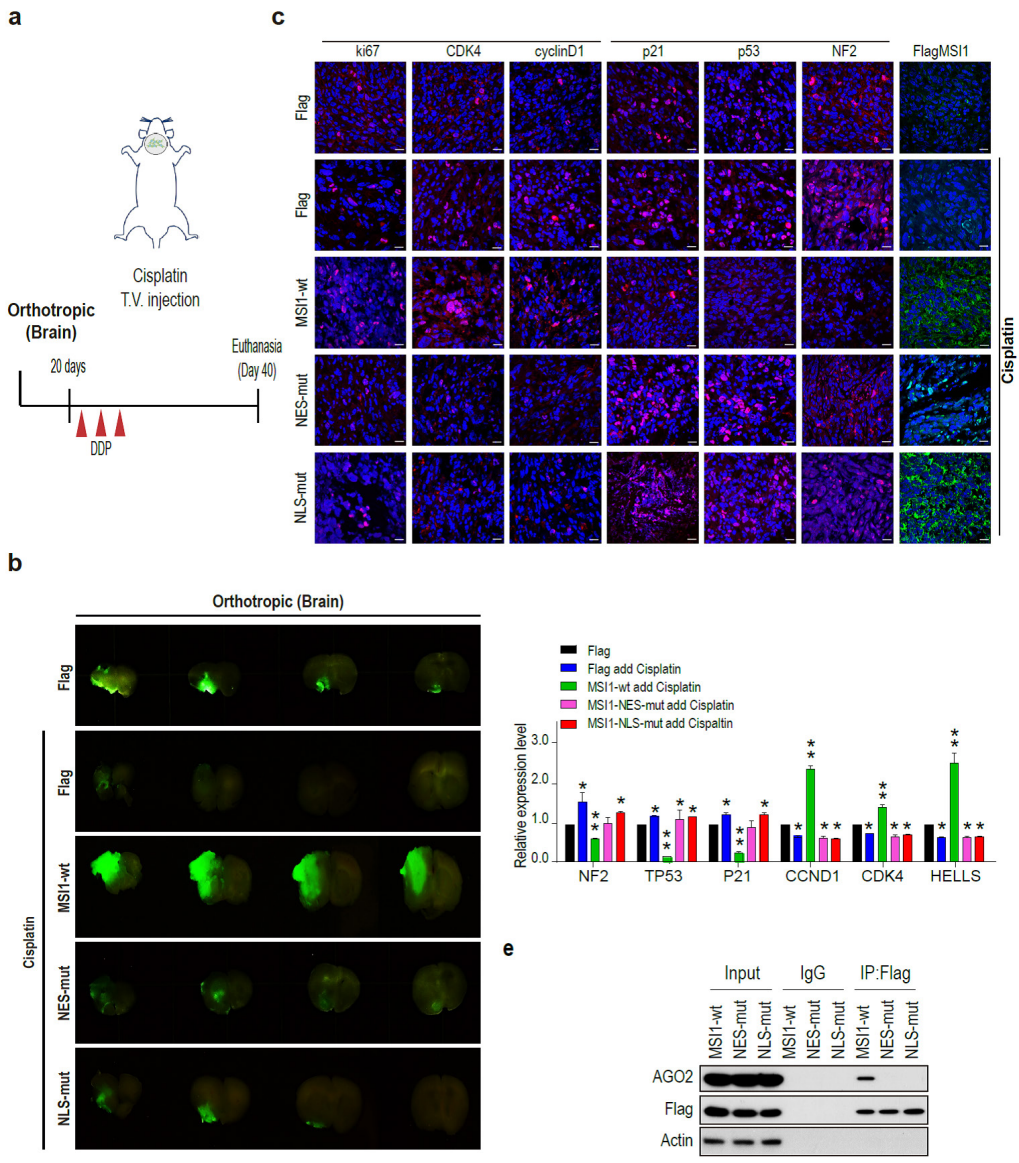
** MSI1 interacts with AGO2 in the cytosol and promote tumor growth. (A)** Schematic illustration presenting the experimental design of xenograft orthotropic tumor model. SCID mice were orthotopically transplanted with 05MG/Flag-control, 05MG/MSI1-wt, 05MG/MSI1-NES-mut, or 05MG/MSI1-NLS-mut GFP cells. Twenty days after transplantation, mice were administered 3 times with 2-day interval of Cisplatin (20 mg/kg) or PBS via tail-vein injection. **(B)** Xenograft tumors were excised 20 days after Cisplatin treatment. Representative images of GFP-positive tumors are shown. N = 6, **P < 0.05. **(C)** Xenograft orthotropic tumor tissue were sectioned and subjected to IHC to evaluate Flag-tagged tumor, NF2, p53, p21, cyclinD1, CDK4 and ki67 expression levels. **(D)** Tumors tissues (five of each group) were harvested and homogenized. Whole-tumor lysates were analyzed by qPCR. (E) Co-immunoprecipitation of endogenous AGO2 with Flag-tagged MSI1 using Flag antibody in 05MG/MSI1-WT, 05MG/MSI1-NES-mut, and 05MG/MSI1-NLS-mut xenograft tumors tissues.

We further affirmed the phenomenon of MSI1-WT, MSI1-NLS-mut, and MSI1-NES-mut overexpression under cisplatin treatment in an in vivo orthotropic xenograft mouse model (Figure 4A). Orthotropic injection of cells overexpressing MSI1-wt combined with cisplatin treatment led to increased size tumor, suggesting that these cells were highly resistant to the cisplatin treatment (Figure 4B). In addition, growth of MSI1-NES-mut and MSI1-NLS-mut tumors was reduced compared to that of the parental cells, suggesting cisplatin sensitivity (Figure 4B). Consistent with our previous results, the protein expression of group 1 genes (*NF2*, *p21*, *TP53*) was decreased and the expression of group 2 genes (*CCND1* and *CDK4)* was upregulated in MSI1-WT tumors after cisplatin treatment (Figure 4C). Moreover, group 1 mRNA targets were downregulated whereas group 2 mRNA targets were upregulated in MSI1-wt tumors.

No changes in the expression of mRNA targets were observed in the MSI1 mutant tumors (Figure 4D). Immunoprecipitation of MSI1 and AGO2 from the orthotropic xenograft tumor tissue confirmed the interaction between MSI1 and AOG2 in the MSI1-wt but not MSI1-NES-mut and MSI1-NLS-mut tumors (Figure 4E). Together, these results indicated that the MSI1-AGO2 interaction and MSI1 translocation capability governs the fate of their downstream targets as well as chemodrug-resistant tumor progression of GBM.

### MSI1/AGO2 binding to a specific location on its targets mediates distinct mRNA fates

**Figure 5:**
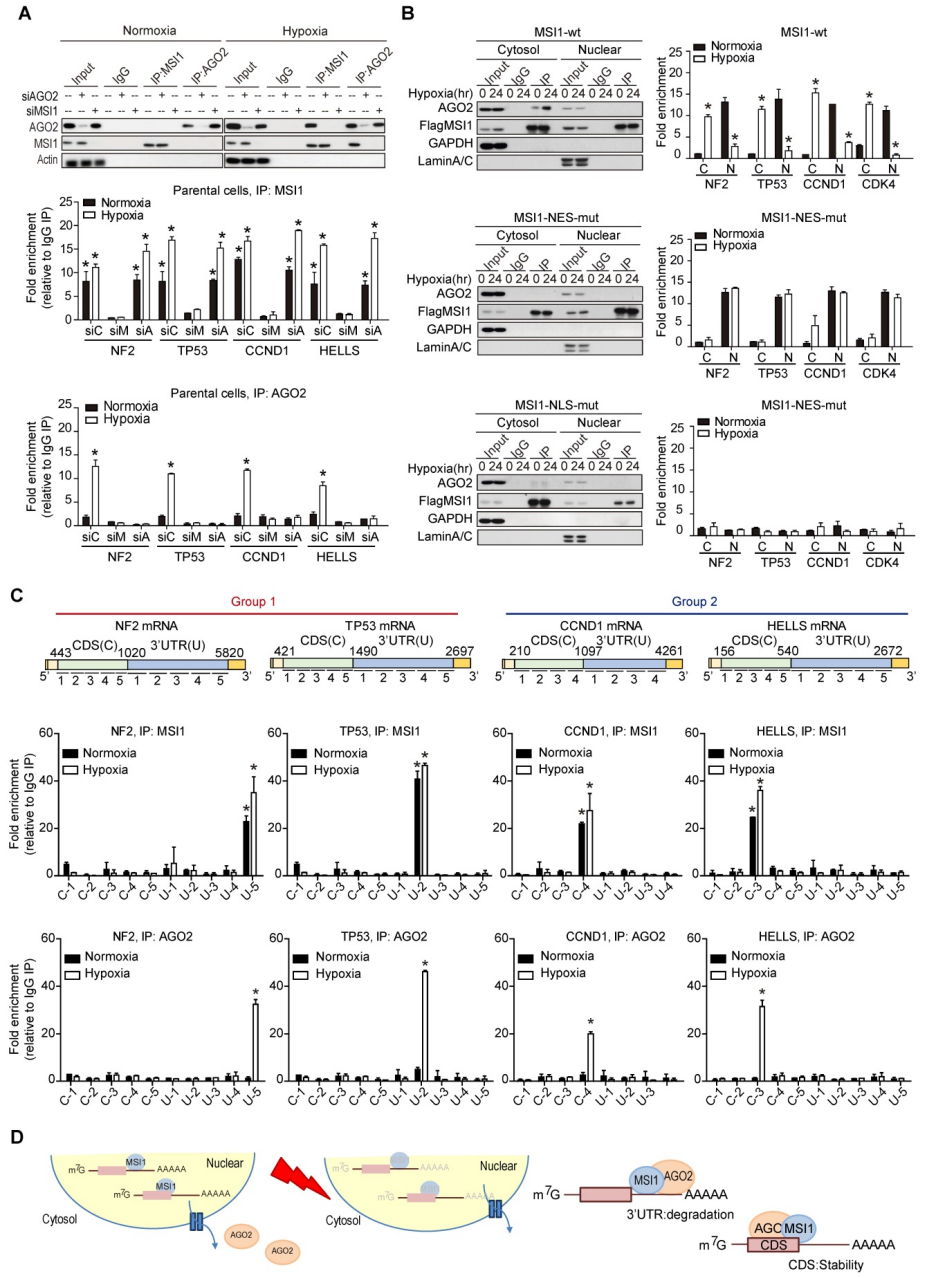
** Differential regulation of mRNAs by MSI1/AGO2 complex under hypoxia. (A)** Endogenous MSI1 or AGO2 was immunoprecipitated in MSI1 or AGO2 knockdown 05MG cell with anti-MSI1 or anti-AGO2 antibody. Western blot of the immunoprecipitation (IP) confirmed the MSI1/AGO2 interaction in hypoxia-treated parental cells but not in MSI1 or AGO2 knockdown cells (top). Total RNAs isolated from IP were subjected to NF2, TP53, CCND1, and HELLS mRNA quantitation by using qPCR with specific primer. Quantification of mRNA expression levels experiments by normalization with IgG control. Data represent the mean ± S.D. of three independent experiments performed in triplicate. * P<0.05 vs IgG signal. **(B)** Nuclear and cytosolic fractions of 05MG/MSI1-wt, 05MG/MSI1-NES-mut, and 05MG/MSI1-NLS-mut cells were subjected to the immunoprecipitation with Flag antibodies to pull down the complexes interacting with Flag-tagged MSI1. Left, immunoprecipitates were subjected to Western blot to assess the binding between AGO2 and full-length or mutated MSI1. Right, total RNAs isolated from the immunoprecipitated complexes were analyzed by qRT-PCR for NF2, TP53, CCND1, and HELLS mRNA levels. Fold change in mRNA levels was normalized to IgG-precipitated controls. Data represent the mean ± S.D. of three independent experiments performed in triplicate. * P<0.05 vs IgG signal. **(C)** Modified-RIP analysis of the binding regions of MSI1 and AOG2 on the target mRNAs. RIP were performed with anti-MSI1 or anti-AGO2 followed by RNA fragmentation and qPCR of NF2, TP53, CCND1, and HELLS coding sequence (CDS) and 3´ UTR. The schematic illustration showed the relative locations of each pair of qPCR primers for CDS and 3'UTR regions. MSI1 or AGO2 palindromic-binding sequence exists within the peak. Quantification of fold changes of the signals were normalized to IgG-precipitated controls. This experiments were done in three distinct biological replicates. **(D)** A schematic illustrating the fate of mRNA determined by the MSI1-AGO2 complex. MSI1-AGO2 regulates RNA stability of specific RNAs to sustain tumor growth under stress in two ways: 1) MSI1-AGO2 facilitates tumor suppressor gene mRNA decay to prevent stress-induced cell death (likely through the conventional UTR binding followed by post-transcriptional repression) and 2) MSI1-AGO2 stabilizes and protects mRNA of cell cycle genes to promote prompt translation upon stress removal (likely through the CDS binding and subsequent aggregation in stress granules).

To decipher the molecular mechanisms by which MSI1/AGO2 complex regulates mRNA stability, we carried out RIP experiments in control, MSI1- and AGO2-knockdown cells under normoxia and hypoxia. Interestingly, MSI1 bound to its mRNA targets under normal and hypoxic conditions while AGO2 bound to its targets only under hypoxia (Figure 5A). Knockdown of AGO2 did not affect MSI1 binding to its mRNA targets whereas knockdown of MSI1 hampered AGO2 recruitment (Figure 5A), suggesting a MSI1-dependent recruitment of AGO2 to mRNA targets under hypoxia. We next investigated the impact of MSI1 shuttling on MSI1-mRNA complex formation. To do so, we performed RIP experiments with the cytosolic and nuclear fractions of cells overexpressing MSI1-wt cultured under normoxia and hypoxia conditions. We showed that under normoxia, MSI1-mRNA complexes were in the nucleus and that upon hypoxia, they were enriched in the cytosol (Figure 5B, top right panel), suggesting an active translocation of MSI1-mRNA complexes into the cytosol in response to hypoxia. When MSI1 failed to shuttle into the cytosol (MSI1-NES-mut), the MSI1-mRNA complex remained in the nuclear compartment as expected (Figure 5B, middle right panel). Surprisingly, the cytosolic mutant of MSI1 (MSI1-NLS-mut) was unable to bind RNA (Figure 5B, bottom panel). Our data suggested that MSI1 first needs to bind its target mRNAs in the nucleus before carrying them into the cytosol in response to hypoxia. Consistently, the recruitment of AGO2 to the MSI1-mRNA complexes occurred in the cytosol (Figure 5B, top left panel). However, MSI1-NLS-mut did not interact with AGO2 (Figure 5B, bottom left panel), suggesting their interaction to be RNA-dependent in the cytosol. We next further characterized binding regions of MSI1/AGO2 complex on its mRNA targets by performing a modified-RIP assay which started with RIP to precipitated MSI1 and AGO2 along with their bound mRNAs, followed by RNA fragmentation and qPCR with region-specific primers (Figure 5C). We showed that, in response to hypoxia, MSI1/AGO2 complex bound the three prime untranslated (3'-UTR) region of target mRNAs from group 1 while it bound the coding sequence (CDS) region of those from group 2 (Figure 5C). Collectively, our data showed that upon hypoxia, MSI1 together with its bound mRNA targets translocate into the cytosol where it subsequently recruits AGO2 to mediate two distinct types of posttranscriptional regulation: degradation of mRNA targets via binding their 3'-UTR (group 1) and stabilization of mRNA targets through binding their CDS (group 2) (Figure 5D).

### Disrupting MSI1/AGO2 interaction restrains tumor growth and alters mRNA regulation

**Figure 6:**
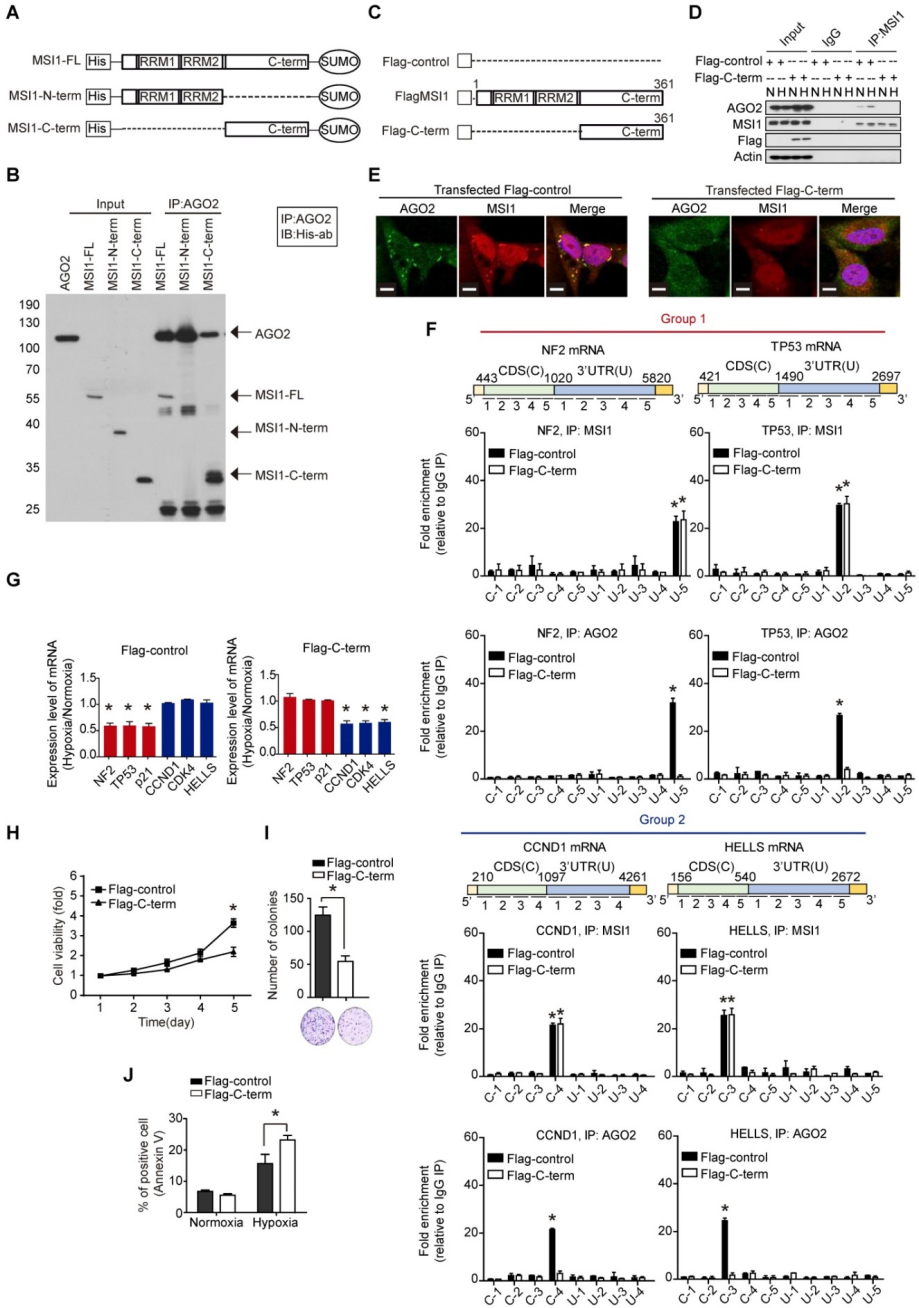
** The C-terminal of MSI1 is critical for AGO2 binding and cell viability. (A)** Schematic presentation of the constructs of full-length, C-terminus, and N-terminus of MSI1 as well as wild-type AGO2 for purifying recombinant proteins. **(B)** Pull-down assay with recombinant MSI1 and AGO2 proteins showed that the C-terminus of MSI1 is essential for the direct MSI1/AGO2 interaction. **(C)** A Schematic illustrating the Flag-control, full-length and C-terminal (C-term) fragment of human MSI1. The construct of MSI1 C-term was sub-cloned into p-3×Flag-Myc-CMV expression vector. **(D)** Cells transient transfected with Flag-control or Flag-tagged MSI1 C-term (Flag-C-term) were subjected to co-immunoprecipitation assay for endogenous AGO2 and MSI1 protein-protein interaction. Transfection of the Flag-C-term blocked hypoxia-induced MSI1/AGO2 binding. **(E)** Cells transfected with Flag-control or Flag-C-term were analyzed under confocal microscopy for the subcellular co-localization of MSI1 (Red) and AGO2 (Green). **(F)** Flag-control and Flag-C-term transfected cells were subjected to an modified-RIP assay using anti-MSI1 or anti-AGO2 antibodies, followed by RNA fragmentation and qRT-PCR analysis to determine the fold change enrichment of the coding sequence (CDS) and 3´ UTR of the NF2, TP53, CCND1 and HELLS mRNAs. Quantification of the fold changes of binding signals was performed by normalizing IP signals to IgG-precipitated controls. The peaks indicated MSI1 or AGO2 palindromic-binding sequence. Flag-C-term blocked the binding of AGO2 but not MSI1 to target sequence in mRNAs. **(G)** Flag-control and Flag-C-term transfected cells were subjected to normoxia or hypoxia for 24 hr. Purified total RNA was subjected to RT-PCR using primers specific for NF2, TP53, CDKN1A, CCND1, CDK4, and HELLS. The mRNA levels under hypoxia were normalized with that under normoxia and presented as relative fold changes in the chart.** (H)** 05MG cells transiently transfected with Flag control or Flag-tagged MSI1 C-term were subjected to an MTT viability assay. The relative fold change of the number of viable cells in each day was presented in the graph. **(I)** Flag-control and Flag-C-term transfected cells were subjected to colony formation assay for 10 days and quantitated by ImageJ software. **(J)** The percentage of apoptotic cells of Flag-control and Flag-C-term transfected cells was determined by external Annexin-V under normoxiac and hypoxic conditions.

As MSI1 engages AGO2 to promote tumor progression through mRNA regulation, we asked whether the disruption of MSI1/AGO2 interaction could affect the tumor growth driven by cytosolic MSI1. To do so, we first mapped MSI1/AGO2 interaction using deletion mutants of MSI1 (Figure 6A). We performed an in vitro binding assay by incubating the purified full-length, the N-terminal or C-terminal domain of MSI1 with purified AGO2 protein, and found that AGO2 preferentially interacted with the C-terminal domain of MSI1 (Figure 6B). We next investigated whether the C-terminal domain of MSI1 (Figure 6C) could act as a decoy to withdraw MSI1/AGO2 protein-protein interaction. Flag control (Flag) or Flag-tagged MSI1 C-terminus (Flag-C-term) were transiently expressed in GBM cells which were then subjected to immunoprecipitation against endogenous MSI1 in normal and hypoxic conditions. We found that overexpression of Flag-C-term disrupted the interaction between endogenous MSI1 and AGO2 (Figure 6D), confirming the importance of the MSI1 C-terminal domain in this protein-protein interaction. Confocal microscopy further confirmed uncoupled co-localization of endogenous MSI1 and AGO2 in cells transfected with Flag-C-term (Figure 6E). To precise the molecular mechanisms underlying the biological effects of Flag-C-term, we performed modified-RIP assay and demonstrated that Flag-C-term interfered with the recruitment of AGO2 to its mRNA targets, at both 3'UTR and CDS regions (Figure 6F). We next analyzed the expression of MSI1/AGO2 mRNA targets and showed that, in cells transfected with Flag-C-term, the expression of mRNA targets from group 1 increased while that of targets from group 2 decreased after hypoxia compared to the control cells (Figure 6G). The protein expression level of MSI1/AGO2 targets was increased in group 1 but decreased in groups 2 (Figure S7A).

**Figure 7:**
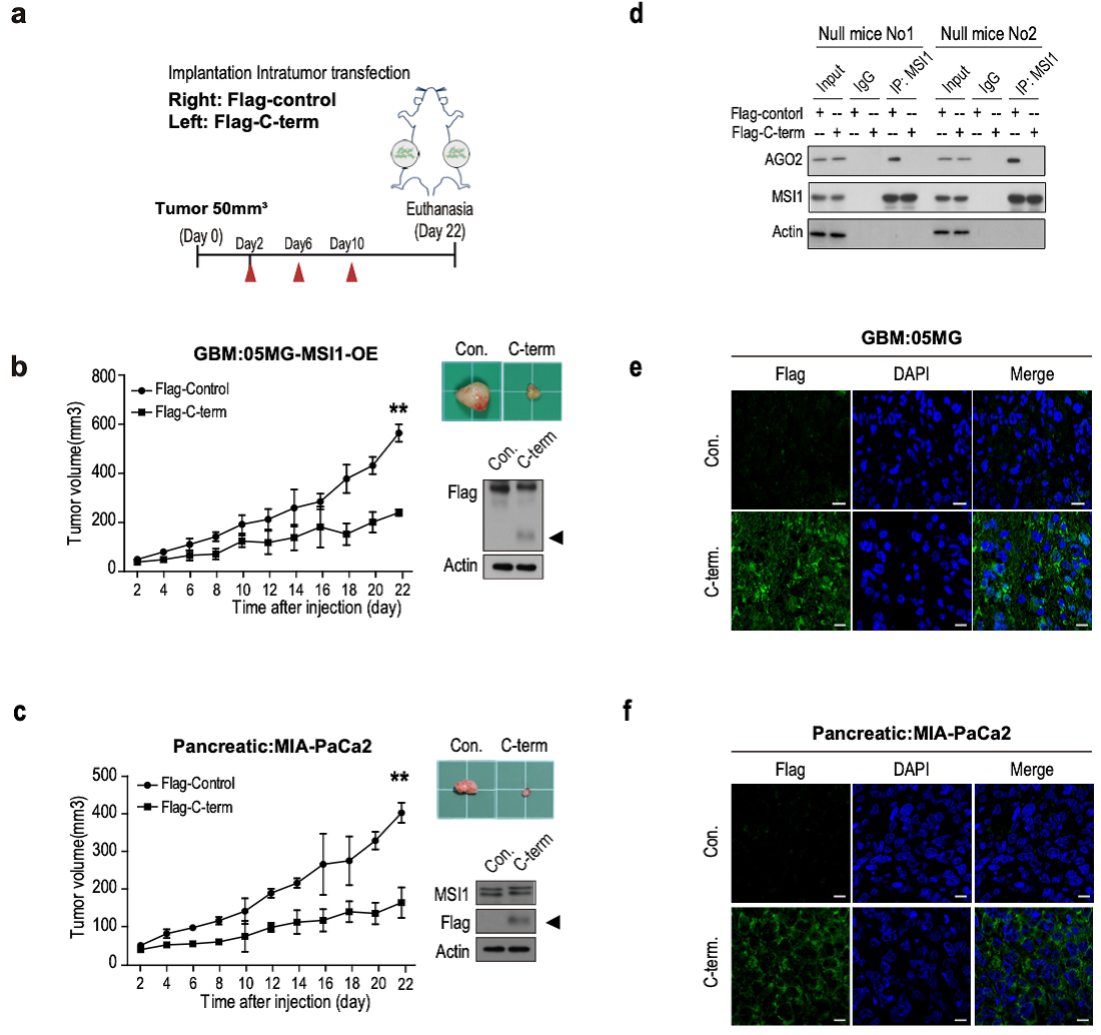
** The C-terminal of MSI1 suppress xenograft tumor growth in GBM and PDAC animal models. (A)** A schematic presentation showing the design of animal experiment with in vivo delivery of Flag-C-term (10 µg using *in vivo*-jetPEI in vivo nucleic acid delivery reagent). Xenograft tumor size was monitored from day 2 after injection of Flag-control or Flag-C-term. **(B)** Immunocompromised mice were subcutaneously transplanted with 05MG/Flag-MSI1 stable cells. Two days after tumor size reached 50 mm^3^, mice were intratumorally injected with 10 µg of Flag-control or Flag-C-term for 3 rounds with 2-day intervals. Tumor size was then monitored for 22 days. The expression of MIS1-C-term in the xenograft tumor tissue was assessed by Western blot. **(C)** Immunocompromised mice were subcutaneously transplanted with MIA-PaCa2 cells. Two days after tumor size reached 50 mm^3^, mice were intratumorally injected with Flag-control or Flag-C-term for 3 rounds with 2 days interval. Tumor size was then monitored for 22 days. The expression of MIS1-C-term in the xenograft tumor tissue was assessed by Western blot. **(D)** Tumor tissue were harvested and homogenized for Western blotting. Whole-tumor lysate were subjected to co-immunoprecipitation assay for endogenous AGO2 and MSI1 protein-protein interaction. Data represent the mean ± S.D. of three independent experiments performed in triplicate. Tumor tissues of **(E)** 05MG/GBM cells and **(F)** MIA-PaCa2/pancreatic cells from MSI1-C-term injected xenografts were immunostained with anti-Flag antibodies to observer the expression of MSI1-C-term in the tumors.

We further evaluated the downstream effects of MSI1/AGO2 complex disruption by analyzing cell viability in cells transfected with Flag or Flag-C-term. Our results showed a decreased percentage of viable cells after Flag-C-term expression (Figure 6H) which is consistent with the decreased number of soft agar colonies (Figure 6I), and the increased percentage of apoptotic cells (Figure 6J). Taken together, these results indicated that, by disrupting MSI1/AGO2 interaction, Flag-C-term suppressed clonogenic growth and promoted apoptosis. To test the therapeutic potent of MSI1 C-terminus, we launched an in vivo study in which cancer cells were implanted on each flank of the mice subsequently subjected to an intratumoral transfection of Flag control or Flag-C-term (Figure 7A). We observed that the growth of tumors derived either from MSI1- overexpressing GBM cells or MIA-PaCa2 pancreatic cancer cells was strongly delayed by the administration of Flag-C-term (Figure 7B-C). Overexpressed C-terminus in tumor cells disrupts endogenous MSI1/AGO2 interaction and reduces tumor volume (Figure 7D-E). Collectively, our findings demonstrated that disrupting MSI1/AGO2 interaction with MSI1-C-term decoy suppressed tumor growth by blocking the recruitment of AGO2 to its target mRNAs and subsequently altering their stability.

### The MSI1/AGO2 pathway is enhanced in patients with tumor relapse

Cytosolic MSI1 engages AGO2 to promote stress-induced tumor growth through RNA regulation. We showed that a significant proportion of MSI1 was cytosolic in samples from patients with high grade glioma (Figure 1A) but it remains unclear whether MSI1/AGO2 pathway actively participates to tumorigenicity in patients and could also have an impact on tumor recurrence. To address this point, we collected eighteen pairs of primary and recurrent GBM samples from patients who received concurrent chemotherapeutics after primary surgery (Tables S2) and analyzed the subcellular localization of MSI1 by IHC staining.

**Figure 8:**
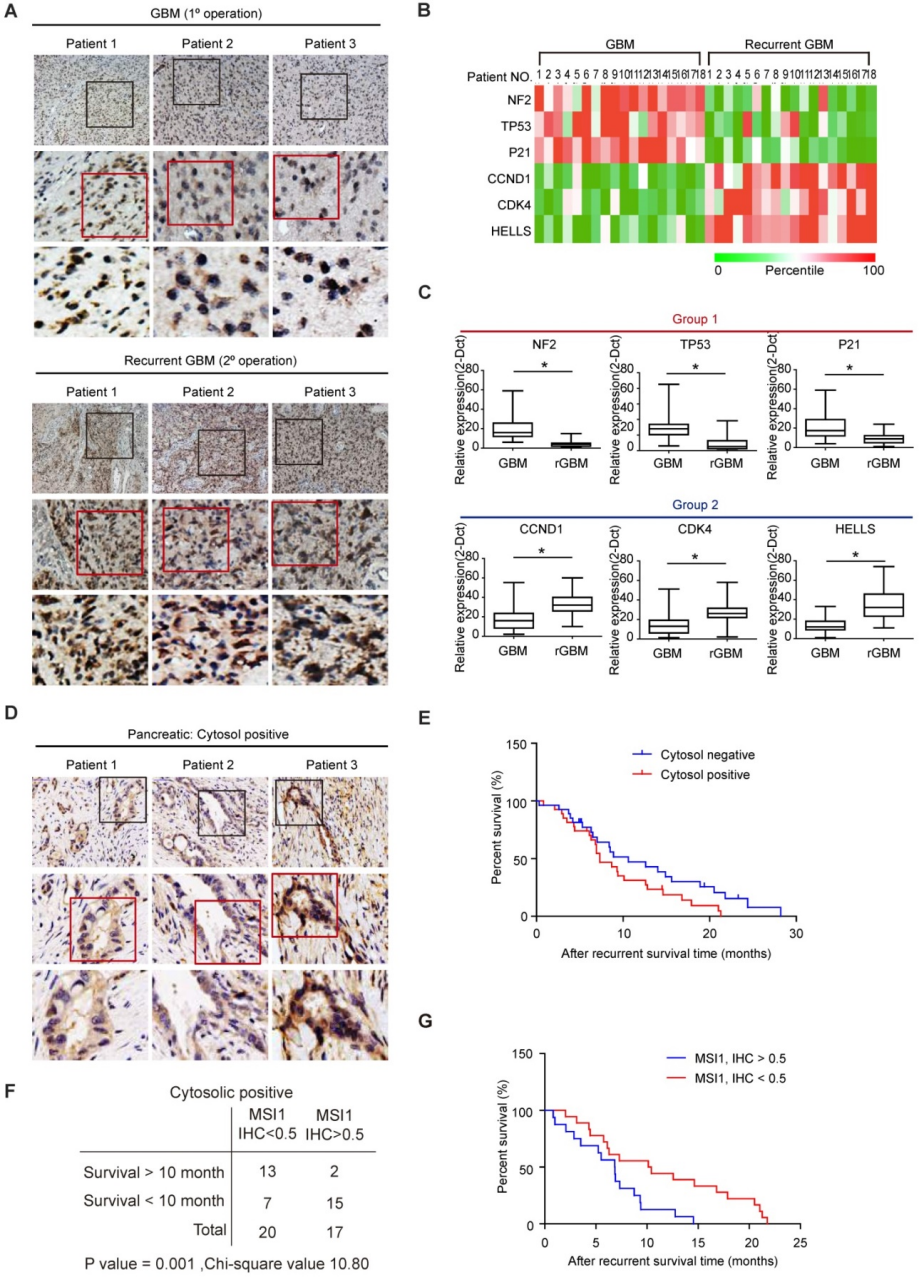
** Cytosolic MSI1 expression associates with GBM relapse and PDAC recurrence in patients. (A)** MSI1 expression was examined by IHC in 18 paired primary and recurrent GBM tissues. Three representative cases (Pt 1 to 3) were presented. Boxes highlighting MSI1 expression pattern. **(B)** qPCR analysis of NF2, TP53, p21, CCND1, CDK4, and HELLS mRNA expression levels in microdissected tumor (T) and stroma (S) samples from the 18 paired primary and recurrent GBM specimens. All mRNA expression levels in T parts were first normalized by that in respective S counterparts, and then the total 36 expression levels (primary and recurrent) of each mRNA were rated as percentile from 0% (green) to 100% (red). A heat map shows the relative mRNA expression levels between paired primary and recurrent GBM tissue. **(C)** qPCR analysis of NF2, TP53, p21, CCND1, CDK4, and HELLS mRNA levels in a group of primary (N = 67) and recurrent (N = 32) GBM tissues (*P < 0.01). P values were estimated by a log-rank test. **(D)** 61 recurrent PDAC patient samples were collected and stained for MSI1 by IHC. 3 representative cases showed positive stain of cytosolic MSI1. **(E)** Survival analysis of the cytosolic MSI1-positive (cytosol-positive; N = 37) and cytosolic MSI1-negative (cytosol-negative; N = 24) recurrent PDAC patients indicates that cytosolic MSI1-positive patients have poorer survival outcome than cytosolic MSI1-negative patients. **(F)** In the 37 cytosol-positive PDAC cases, the expression level of cytosolic MSI1 were evaluated by IHC score. In the 20 cases with cytosolic MSI1 IHC score < 0.5, 13 cases survived over 10 months after recurrence; while in the 17 cases with cytosolic MSI1 IHC score > 0.5, only 2 cases survived over 10 months after recurrence. P = 0.001; Chi-square = 10.80. **(G)** Post-recurrent survival analysis of the two groups (IHC score > 0.5 and IHC score < 0.5) of cytosol-positive PDAC patients.

We showed that a significant proportion of MSI1 proteins were cytosolic in the GBM recurrent samples whereas MSI1 was barely detectable in the cytosol in the primary GBM samples (Figure 8A). We then collected by laser capture microdissection (LCM) tissue samples from the tumor (T) and non-tumor stroma (S)^28^ and analyzed the expression level of MSI1 target mRNAs by qPCR. We observed that, for each pair of samples, the expression of mRNA targets related to apoptosis (group 1) was decreased in recurrent GBM compared to the primary GBM while the expression of the mRNA targets related to cell cycle (group 2) was increased (Figure 8B). The same results were also observed in a follow-up of a cohort of primary (n = 67) and recurrent (n = 32) GBM patients (Figure 8C and Table S3). These results indicated that, in patients with recurrent GBM, the MSI1/AGO2 pathway was enhanced to promote tumor growth and ensure cancer cell survival.

To address whether the correlation between MSI1/AGO2 pathway and tumor recurrence could be generalized to other cancer types, we collected samples from patients with pancreatic ductal adenocarcinoma (PDAC) and performed IHC staining of MSI1 on non-recurrent (n = 18) and recurrent (n = 61) PDAC samples (Table S4). We observed that around 5% of non-recurrent pancreatic samples exhibited MSI1 in the cytosol (1/18 cases; data not shown) while 60% of recurrent PDAC samples (37/61 cases) displayed cytosolic MSI1 (Figure 8D), suggesting that cytosolic MSI1 was associated with tumor recurrence. By analyzing clinical data of the recurrent PDAC samples, we observed that patients with recurrent PDAC positive for cytosolic MSI1 presented overall a lower survival than those negative for cytosolic MSI1 (Figure 8E). We further analyzed the 37 recurrent PDAC cases positive for cytosolic MSI1 and classified them based on IHC staining score (IHC<0.5 or IHC>0.5). We showed that the group of patients with high score (IHC > 0.5) exhibited a lower survival outcome than that with low score (IHC < 0.5) (Figure 8F-G), suggesting that the level of cytosolic MSI1 could predict patient survival. Collectively, our results indicated that the cytosolic MSI1/AGO2 complex interaction and downstream pathway was significantly enhanced in patients with tumor relapse and could affect patient survival.

## Discussion

GBM and PDAC are highly malignant tumors with dismal prognosis and a short survival time even after a completed treatment 28, 29. Environmental stresses play a pivotal role in both stem cell maintenance and tumorigenesis.

Here we reported that MSI1 shuttles into the cytosol under stress to form a complex with AGO2 that can stabilize or destabilize its target mRNAs. Sutherland and colleagues reported in 2017 that MSI2 translationally repressed *Piwil1,* another member of the Argonaute protein family, during male gamete development 30, directly linking musashi proteins with the Argonaute family. In our study, we identify the MSI1/AGO2 interaction by proteomic analysis from immunoprecipitation in cancer cells (Figure S4B-C). It seems to us that the two proteins did not regulate the protein expression levels of each other as knockdown of MSI1 did not change the protein expression of AGO2, neither did vice versa (Figure 2E and 2G). In the case of MSI1 and AGO2, the two proteins work together to determine the fate of their downstream RNA targets.

Cytosolic MSI1 enhances tumor proliferation and cancer cell survival through this AGO2-dependent mRNA regulation. AGO2 is essential for MSI1 pro-tumor progression as its knockdown 31 inhibited the MSI1-enhanced tumor growth (Figure S4F). Intact functions of MSI1 shuttling are essential to drive the tumorigenicity (Figure 1D-L). The sequestration of MSI1 in the nucleus by disrupting the NES resulted in lack of its mRNA regulatory functions while surprisingly, constitutively cytosolic MSI1 (NLS mutant) also lost its ability to regulate mRNA targets. Indeed, MSI1 NLS-mutant does not bind RNA in vivo (Figure 5B), suggesting a model in which MSI1 must first bind its mRNA targets in the nucleus to then relocate them into the cytoplasm. The RNA binding and sub-cellular translocation of RNA-binding proteins may be regulated by post-translational modifications (PTMs)^32^, ^33^. We demonstrated that MSI1 acts as a guide to recruit AGO2 and subsequently regulate the stability of their target mRNAs. The MSI1/AGO2 complex binds mRNAs including genes involved in apoptosis and cell cycle progression, but exhibits two distinct types of regulation. MSI1/AGO2 complex degrades mRNA targets by binding to their 3'UTR while it stabilizes mRNA targets by binding to their CDS (Figure 5). How can MSI1/AGO2 control these two distinct modes of mRNA regulation? Target mRNAs that are not engaged in translation aggregate into cytosolic organelles such as stress granules (SG; for RNA stabilization) and Processing-bodies (P-bodies; for RNA degradation) 34-38. AGO2 has been reported to remodel its interactions with target mRNAs and regulate their post-transcriptional control under stress responses 23, 39. In response to stress, AGO2 could remodel its occupancy on the 3'UTR and CDS of target mRNAs and then adjust the translation rate of specific group of genes.

Although how AGO2 coordinates its functions in response to stresses is still unclear, our findings highlighted a sophisticated mechanism by which MSI1/AGO2 complex promote tumor progression. In response to stress, the complex stabilizes a subset of mRNAs related to cell cycle to promote tumor progression but also regulate the stability of another subset of mRNA targets related to apoptosis to ensure cancer cell survival. Interestingly, MSI1 and AGO2 could also have independent mRNA regulatory functions. We identified other subsets of genes that are only regulated by either MSI1 or AGO2 (Figure 3A). These distinct regulations could rely on different protein-protein interactions. For example, we showed that MSI1 is able to specifically interact with other proteins in response to hypoxia (PABPC1, PKR, GCN2, eIF3A, eIF2C2) (Figure S4B-C) that could play a role in a different type of regulation. Moreover, we showed that AGO2 specifically associates with the C-terminal region of MSI1. No crystal structure of this region exists and our in silico simulation indicated that the C-terminus of MSI1 is an intrinsically disordered region that has no specific structure and is very flexible. We demonstrated* in vitro* and *in vivo* that C-terminal terminus of MSI1 could work as a decoy to disrupt the endogenous MSI1/AGO2 interaction, and to efficiently block the oncogenic functions of the endogenous MSI1/AGO2 complex as well as inhibit xenograft tumor growth (Figure 7).

**Figure 9:**
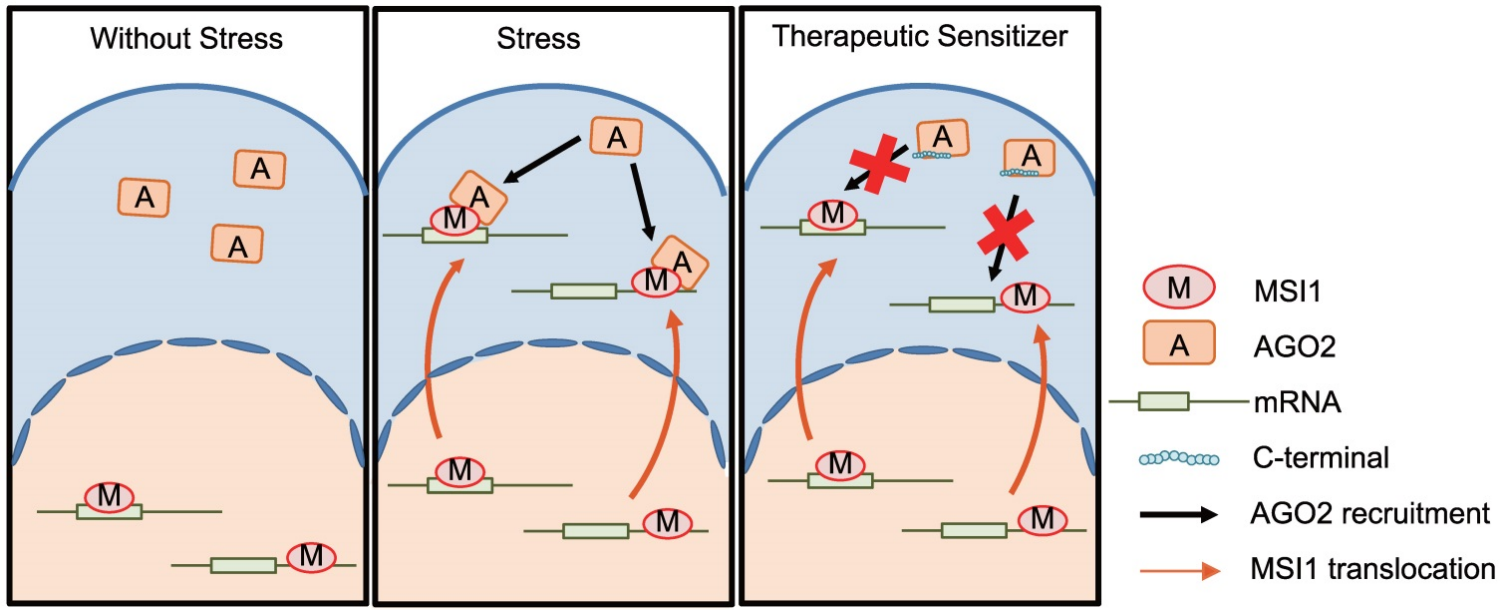
Under stress conditions, MSI1 translocates to cytosol and binds to AGO2 to promote tumorigenesis. Blocking the MSI/AGO2 interaction with MSI1 C-terminus decoy suppress MSI1-dependent drug resistance and tumor progression.

MSI1 is not the only RNA-binding protein that is predominantly nuclear and can shuttle to the cytoplasm upon environmental stress stimulation. The RBM45 (RNA-Binding Motif 45) protein translocates from nuclear to accumulate into the cytosol in Amyotrophic Lateral Sclerosis (ALS) patients 40. This shuttling of RBM45 is used as a pathological marker for the disease 40. Cytosolic MSI1 enhances tumor proliferation and cancer cell survival, and highly correlates relapse occurrence in patients with GBM and PDAC (Figure 9). We could use MSI1 subcellular localization as a biomarker to further improve the diagnosis and prognostic of GBM and PDAC. More importantly, significant efforts should be put in the future on understanding the mechanisms of MSI1 shuttling (hypothesis of specific PTMs or protein-protein interaction for example). The development of drugs that can prevent MSI1 translocation from the nucleus into the cytosol represent a promising therapeutic approach to decrease GBM and PDAC relapse incidence after chemotherapy. This study offers an alternative possibility to decrease GBM and PDAC tumor progression by using a peptide-based approach. The disruption of MSI1/AGO2 interaction with decoy peptides efficiently inhibits tumor growth, suggesting that peptide-based therapy could be of importance to decrease recurrent GBM and PDAC. Similar therapeutic strategies are already developed by other groups. Chang et al. used a stapled α-helical peptide to efficiently abolish p53/MDM2 interaction and induce the death of cancer cells 41. Notably, overexpression of MSI1 C-terminus increased sensitivity to chemotherapeutic drugs, thus hindering the malignant progression of GBM, which opens the possibility to treat tumor recurrence via tackling the MSI1/AGO2 complex formation.

## Supplementary Material

Supplementary methods, figures, and tables.
