# Supplementary material for: Musashi-1 promotes stress-induced tumor progression through recruitment of AGO2
Source: Theranostics. 2020 Jan 1;10(1):201–17. doi: 10.7150/thno.35895 (PMC6929620; doi:10.7150/thno.35895)
Supplement: Supplementary file 1 — Supplementary methods, figures, and tables. [file thnov10p0201s1.pdf]

SUPPLEMENTARY INFORMATION

FIGURE SLEGENDS

Figure S1. MSI1 is important for tumor malignance and tumor progression.

(A-B) Non-tumorous and different grade of brain tumor tissues from clinical patients were analyzed by IHC to assess the MSI1 expression levels. The ratio of MSI1 expression in each group was presented in the graph. Data are presented as the mean ± SD of triplicated experiments. **p* < 0.001 (Student's t-test). **(C and G)** 05MG cells stably transfected with the Flag-control, Flag-tagged MSI1 or scramble-control and MSI1-knockdown cells. The immunoblots to confirm the protein expression of Flag-tagged MSI1 or endogenous MSI1 with anti-flag antibody or anti-MSI1 antibody. **(D and H)** 05MG/Flag-control and 05MG/Flag-tagged MSI1 or scramble-control and MSI1-knockdown cells were subjected to colony formation assay for 10 days, and the numbers of colony were quantitated by Image J software. **(E and I)** 05MG/Flag-control and 05MG/Flag-MSI1 or scramble-control and MSI1-knockdown cells were subjected an apoptosis assay determined by annexin V staining. **(F and J)** Null mice were subcutaneously transplanted with 05MG/Flag-control and 05MG/Flag-tagged MSI1 or scramble-control and MSI1-knockdown cells. Tumor size was measure with a caliper at the indicated time points. N = 6. **P < 0.05 vs. 05MG/Flag-control cells or 05MG/Scramble cells.

Figure S2. MSI1 translocated into cytosol under hypoxia or cisplatin treatment in different cell lines.

(A-D) Left: Immunoblots of the protein expression of MSI1, Laminin A/C and GAPDH in nuclear and cytosolic compartments of two patient-derived primary GBM cells (Pt 3 and Pt 11) as well as MIA-PaCa2 PDAC cells treated with 24-hour hypoxia (T: total protein, C:

cytoplasmic, N: nuclear). Right: Pt 3, Pt 11, and MIA-PaCa2 under normoxia or hypoxia for 24 hours were subjected to anti-MSI1 (green) immnunofluorescent staining and DAPI (blue) nuclear counter stain. Images were acquired from Carl Zeiss confocal microscope system. Both cells showed increased cytosolic MSI1 under hypoxia condition. **(E)** The translocation of MSI1 in 05MG cells is induced by cisplatin. Top: 05MG cells pre-treated with or without nuclear export inhibitor leptomycin B (LMB) (10 ng/mL, 2 hours) were further treated by cisplatin (30 μM) for 24 hours. The localization of MSI1 and the nucleus were stained by anti-MSI1 (green) and DAPI (blue), respectively. **(F)** Functional validation of the identified nuclear exporting signal (NES) using GFP-tagged expressing vectors. Cells transfected with the GFP, NES fused GFP (NES-wt-GFP), or GFP fused with mutated NES (NES-mut-GFP) were treated with or without LMB (10 ng/ml for 2 hours). Top: The scheme for the construction of wildtype NES and mutated NES fused with GFP protein; Bottom: the confocal microscopy imaging for the GFP distribution. **(G-I)** 05MG cells transfected with wildtype or mutated MSI1 were subjected to functional analyses to assess cell proliferation, apoptosis, and clonogenic growth. Hypoxia-induced apoptosis was withdrawn by the over-expression of wild-type MSI1 but not in mutant MSI1 groups determined by annexin V staining, MTT assay, and colony forming assay.

Figure S3. The trafficking MSI1 is essential in MSI1-mediated oncogenic events.

05MG cells stably transfected with the Flag-control, Flag-tagged MSI1-wt, Flag-tagged-MSI1-NES-mut and Flag-tagged MSI1-NLS-mut cells. **(A)** The immunoblots to confirm the protein expression with anti-flag antibody. **(B)** The real-time PCR to confirm the MSI1 mRNA expression level. **(C)** Total lysate, nuclear, and cytoplasmic fractionations of 05MG cells with different stable clone cells under normoxia (N) or hypoxia for 24 hr (H) were subjected to immunoblotting with Flag, Lamin A/C (nuclear internal control) and GAPDH (cytosolic control) antibodies. **(D)** The different stable clone cells were subjected to an MTT viability assay. **(E)**

The different stable clone cells were subjected an apoptosis assay determined by annexin V staining. **(F)** The different stable clone cells were subjected to colony formation assay for 10 days, and the numbers of colony were quantitated by ImageJ software.

Figure S4. MSI1 interacted with AGO2 in the cytosol under hypoxia and cisplatin treatment.

(**A**) Coomassie blue stained SDS-PAGE of the normoxia and hypoxia samples for LC-MS/MS. **(B)** The list of MSI1-bound and stress-related proteins identified by LC-MS/MS analysis in 05MG cells. **(C)** Immunoblotting confirmed the candidates identified by proteomic analysis with or without RNase A treatment for 1 hr before immunoprecipitation **(D)** Co-immunoprecipitation of endogenous AGO2 with MSI1 in MIA-PaCa2 PDAC cell line treated with or without hypoxia for 24 hours. **(E)** Endogenous AGO2 was immunoprecipitated in 05MG cell lysates with anti-MSI1 antibody under cisplatin (30 μM) stimulation for 24 hrs. MSI1 was pulled down by MSI1 antibody and then subjected to immunoblotting using anti-MSI1 and anti-AGO2 antibody. **(F)** 05MG cells expressing FRET pairs of MSI1-orange and AGO2-GFP were bleached (bottom) at the region of interest (ROI) indicated by the rectangular. Unbleached controls were also performed (top). Fluorescent emission intensities of MSI1 (red) and AGO2 (green) during acceptor photobleaching experiments were shown in the left panel and quantified in the right panel. Quantification of FRET photobleaching experiments was performed by calculating FRET efficiencies for the FRET pairs MSI1 (red)-AGO2 (green). Data represent the mean ± S.D. of three independent experiments performed in triplicate. * $P<0.05$ and ** $P<0.01$ vs control. **(G-H)** 05MG cells were under hypoxia or cisplatin (30 μM) for 24 hrs with or without LMB (10 ng/mL). Co-localization of MSI1 (green) and AGO2 (red) was observed by confocal microscopy. Images were acquired from Carl Zeiss confocal microscope system.

Figure S5. Both MSI1 and AGO2 are essential in MSI1-mediated oncogenic events.

(A-C) The 05MG/Flag-control, 05MG/MSI1-wt, 05MG/MSI1-NES-mut and 05MG/MSI1-NLS-mut cells were subjected to immunoblotting with AGO1, AGO2 and Flag antibodies and real-time PCE to ensure the AGO1 an AGO2 mRNA expression level. (**D**) Western blot analysis confirmed the knockdown efficiency of AGO2 (clone #1 and #2) in MSI1-overexpressed cells. (**E**) 05MG/Flag-control, 05MG/MSI1-wt and 05MG/MSI1-wt/shAGO2 cells were subjected to an apoptosis assay determined by annexin V staining. Hypoxia-induced apoptosis was withdrawn by the over-expression of MSI1 but not with additional knockdown of AGO2. (**F**) Immunocompromised mice were subcutaneously transplanted with 05MG/MSI1-wt and 05MG/MSI1-wt/shAGO2 cells. Tumor size was then monitored for 22 days. N = 6. *P < 0.05 vs. 05MG/MSI1-wt cells.

Figure S6. Identification of mRNA binding targets of MSI1/AGO2.

(A) Total RNAs isolated from immunoprecipitation (IP) in cell under normoxia or hypoxia were subjected to mRNA quantitation by using qPCR with specific primer.

Figure S7. The C-terminal of MSI1 suppress downstream protein expression.

(A) Flag-control and Flag-C-term transfected cells under nomoxia, hypoxia for 24 hrs or recovery to normoxia for 6 hrs condition were subjected to immunoblotting with antibody of TP53, NF2, CDKN1A, CCND1, CDK4, HELLS and actin.

SUPPLEMENTARY METHODS

Cell culture and clinical tissue

The human GBM cell line 05MG (Denver Brain Tumor Research Group 05), human pancreatic ductal adenocarcinoma cell line (MIA-PaCa2), and its derivative stable cell lines, MSI1-WT, MSI1-NES-mut and MSI1-NLS-mut stable cell lines were cultured in Dulbecco's Modified Eagle's Media (DMEM, Life Technologies Inc., Carlsbad, CA, USA) supplemented with 10 % fetal bovine serum (HyClone Laboratories Inc., South Logan, UT, USA), 150g/mL G418 (SIGMA, Cat#A1720), 100 units/mL penicillin, and 100 μg/mL streptomycin (Life Technologies Inc., Carlsbad, CA, USA) under standard culture condition (37℃, 95 % humidified air and 5 % CO2). Sub-cultures were performed with 0.25% trypsin-EDTA (Sigma-Aldrich Co. LLC., St. Louis, MI, USA). All cells lines were tested for microplasma contamination. The clinical tissue samples and tumor cell cultures were acquired from the Neurological Institute of Veterans General Hospitals and Department of Neurological Surgery of Tri-Service General Hospital. All procedures of tissues acquirements have followed the tenets of the Declaration of Helsinki and are reviewed by Institutional Review Committee at Taipei Veterans General Hospital and Tri-Service General Hospital.

Animal care, tumor cell transplantation, and non-invasive imaging

All procedures involving animals were performed in accordance with the institutional animal welfare guidelines of Taipei Veterans General Hospital. For subcutaneous transplantation, cells were harvested, washed, suspended in PBS. A total volume of 100 μl with 1×10^6 cells were injected subcutaneously into the dorsolateral side of the flank region of 8-week-old male BALB/C nude mice (National Laboratory Animal Center, Taipei, Taiwan) bred and maintained following to the Guidelines for Laboratory Animals in the Taipei Veterans General Hospital. Fourteen days after subcutaneous injection, 2 mg/kg of cisplatin was injected mice

twice a week for two weeks through tail vein injection to mimic clinical chemotherapy. Tumor size was measured with calipers [1]. Six mice was used for each condition in each experiment.

For orthotropic transplantation, cells were harvested, washed with PBS, andspindown to remove excess PBS. A total volume of 2.5 μl with 5 × 10^5 were injected orthotropic into the brain of 8-week-old male SCID mice (National Laboratory Animal Center, Taipei, Taiwan) bred and maintained according to the Guidelines for Laboratory Animals in the Taipei Veterans General Hospital. After 14 days of subcutaneous injection, 2 mg/kg of cisplatin was injected mice twice a week for two weeks through tail vein to mimic clinical chemotherapy.

Plasmid constructions and transfection

MSI1 gene were amplified and sub-cloned from human genomic DNA. The p3XFlag-MSI1 and pmOrange-MSI1 plasmids were generated by inserting a 1038-bp fragment of full-length human MSI1 cDNA into the HindIII/BamHI site of p3XFlag-myc-CMV-26 vector (Sigma, No. E 6401) and pmOrange vector (Clontech, No. 632592). PCR amplified DNA fragment with proper restriction cutting sites were introduced by PCR. The primers used for amplification were listed in Table S 5. MSI1-NES-mutant and MSI1-NLS-mutant [2] clones were created by site-directed mutagenesis according to the manufacturer's instruction (QuikChange II site-directed mutagenesis kit, #200523/200524). The used primers were listed as Table S5

MSI1 C-terminal deletion clone was created by PCR amplification using p3XFlag-MSI1 as the template. The DNA fragments were introduced by an additional restriction enzyme cutting sites by PCR. The 3xFlag-MSI1-C-term plasmid or pEGFP-MSI1-C-term were generated by inserting a 539-bp fragment into the p3XFlag-myc-CMV-26 vector or pEGFP-C1-Vector (Clontech, No.632592). The used primers were listed in Table S.5. MSI1 C-

terminal truncation clones were created by PCR amplification using p3XFlag-MSI1 plasmid as the template. The 3xFlag-MSI1-deletion plasmids were generated by inserting 870, 804, 770 and 732-bp fragment into the HindIII/BamHI sites of the p3XFlag-myc-CMV-26 vector. The used primers were listed in Table S5.

In vitro plasmid transfection was carried out using jetPEI DNA transfection reagent (Polyplus Transfection, Huntingdon, UK) according to the manufacturer's instruction. In vivo plasmid transfection in mice were performed with *in vivo*-jetPEI in vivo nucleic acid delivery reagent (Polyplus-tranfection, Illkirch, France). For each intratumoral transfection, 10 μg of FLAG-C-term expression plasmid were mixed with 2 μl of *in vivo*-jetPEI in a total volum of 50 μl.

Gene expression analysis.

The RNA samples from 05MG cells were isolated using TRIzol and confirmed by NanoDrop ND-1000. The RNA integrity was assessed by agarose gel electrophoresis. The gene Expression array (Agilent Technologies) is a customized design with 336 genes identified from the NGS data. The quantitative results were initially aligned by bowtie-1.1.2 and the express-1.5.1 was used for the calculation of quantitative performance following the previous reports [3, 4]. The highest measure of transcripts in average are considered the gene expression, and subsequently standardized by (expression – mean value) / standard deviation.

Gene silencing using small interference RNA (siRNA)

Targeted gene silencing for MSI1, AGO2, and scrambled control were purchased from GE Dharmacon On-TARGETplus siRNA smart pools. Transient transfection was carried out using INTERFERin siRNA transfection reagent (Polyplus Transfection, Huntingdon, UK) according to the manufacturer's instruction (siRNA for MSI1: SASI_Hs01_00145278, siRNA for AGO2: SASI_Hs01_00161740, siRNA for NC cont: SG00217942, Sigma Aldrich Co., St.

Louis, MO, USA). Cell-based experiments were performed after 2-day incubation.

Cell viability assay

MSI1-WT, MSI1-NES-mut, MSI1-NLS-mut and MSI1-C-term in MSI1-overexpressed cells were seeded in 24-well plates (3000 cells per well) with complete growth medium. The medium was replaced by either solvent or chemicals with indicated concentrations in complete medium. Cell viability assay was then performed. In brief, cells were stained with 0.1 mg/ml 3- (4,5-cimethylthiazol-2-yl)-2,5-diphenyl tetrazolium bromide (MTT, SIGMA, Cat#M2003) for 2 hours and the formazon crytals were then dissolved in DMSO. The relative absorbance was then measured by TECAN Sunrise ELISA plate reader (Thermo Scientific Inc., Waltham, MA, USA) at 570 nm light absorbance.

Colony formation assay

MSI1-WT, MSI1-NES-mut and MSI1-NLS-mut were seeded in 6-well plates (1,000 cells per well) and were incubated for 24 hours. The cells were then subjected to hypoxic condition for additional 24 hours. Further 10-day incubation was performed, and the cells were fixed by 10 % formalin, and stained by 4 % trypan blue (w/v) for 20 min. The stained colonies were washed by PBS and counted.

Determination of apoptosis

Apoptotic events were determined by Annexin V (BD Pharmingen™, #556547). For flow cytometry, cells were harvested and stained with both Annexin V and PI for 10 min. The cells were washed by PBS and resuspended in HEPES for subsequent flow cytometry analysis.

Preparation of nuclear and cytosolic extracts

Nuclear and cytosolic extracts were isolated with a Nuclear and Cytoplasmic Extraction kit

(Pierce Chemical, Rockford, IL). After the incubation period, cells were collected by centrifugation at 1000rpm for 5 mins at 4°C. The pellets were washed twice with ice-cold PBS, followed by the addition of 0.2 ml of cytoplasmic extraction buffer A and vigorous mixing for 15 sec. Ice-cold cytoplasmic extraction buffer B (11μl) was added to the solution. After vortex mixing, nuclei and cytosolic fractions were separated by centrifugation at 13000 rpm for 5 mins. The cytoplasmic extracts (supernatants) were stored at -80°C. Nuclear extraction buffer was added to the nuclear fractions (pellets), which were then mixed by vortex mixing on the highest setting for 15 sec. The mixture was chilled, and a 15 sec vortex was performed every 10 mins for a total of 40 mins. Nuclear fraction was centrifuged at 13,000 rpm for 10 mins. The nuclear extracts (supernatants) were stored at -80°C until use.

Western blotting

Protein samples were prepared with RIPA buffer (Thermo Scientific Inc., Waltham, MA, USA) containing 1% protease inhibitor. Equal weight of total protein was separated by electrophoresis on SDS/PAGE. After the proteins had been transferred onto a polyainylidene difluoride membrane (Millipore, Bedford, MA, USA), the blots were incubated with blocking buffer (1 X PBST and 5% skim milk) for 1 hour at room temperature and then hybridized with primary antibodies overnight at 4°C, followed by incubation with horseradish peroxidase-conjugated secondary antibody for 1 hour at room temperature. The blots were obtained by X-ray film exposure, and the intensities were quantified by densitometry analysis (Digital Protein DNA Imagineware, Huntington Station, NY). All antibodies were followed: Table S 8.

RNA extraction

Cells were lysed by TRIzol reagent (Life Technologies Inc., Carlsbad, CA, USA) followed by phenol: chloroform purification and ethanol precipitation. Single strand cDNA was reversely

transcribed by SuperScript III reverse transcriptase (Life Technologies Inc., Carlsbad, CA, USA). Oligonucleotides used for PCR analysis (Table S 6) were designed using Primer Express 2.0 (Applied Biosystems, Foster City, CA, USA).

Quantitative real-time PCR (qRT-PCR)

Oligonucleotide specificity was computer tested (BLAST, National Center for Biotechnology Information, Bethesda, MD, USA) by homology search with the human genome and later confirmed by melting curve analysis. The qRT-PCR was performed with power SYBR Green PCR Master Mix (Applied Biosystems, Foster City, CA, USA) according to manufacturer’s instruction. Signals were detected using 7900HT Fast Real-time PCR system (Applied Biosystems, Foster City, CA, USA). The expression level of each gene was normalized to endogenous 18S and experimental control through ∆Ct methods. All the antibodies and PCR primers used were listed (Table S 6). The heatmap of qPCR array data was visualized in R statistical language with ggplot2 package.

Co-immunoprecipitation (Co-IP)

The cells were washed three times with ice-cold PBS and collected by trypsinizing. After centrifugation, cell pellets were resuspended in Buffer-G (50mM Tris pH 7.5 , 170mM NaCl, 13mM MgCl2, 0.5% NP40, 0.3% Triton X-100, protease inhibitor cocktail) containing 100000 U of RNasin Plus RNase inhibitor (Promega Inc., Waltham, MA, USA, N2615). Firstly, the Dynabeads Protein-G (Invitrogen Inc., Carlsbad, CA, USA, 10003D) was incubated with 2.5μl antibody 30 minutes at room temperature. Next, 1 mg protein lysate was incubated with protein-G conjugated-antibody beads for 6 hours or overnight at 4℃. Dynabeads Protein-G was separated by magnetic beads separation stand (Invitrogen Inc., Carlsbad, CA, USA) and wash 3 times in buffer G. Protein was analyzed by SDS-PAGE. All the used antibodies were listed in Table S 8.

Recombinant proteins and pull-down assay

The cDNA of human AGO2 and MSI1 were obtained from Addgene, PCR-amplified, and subcloned into pFASTBAC vector in-frame to an N-terminal 6xHis or FLAG tag, respectively. The baculoviruses for His-AGO2 and FLAG-MSI1 were prepared according to the manual of Bac-To-Bac Baculovirus Expression System (Thermo Fisher Scientific). Briefly, recombinant Bacmid DNA were isolated from pFastbac-HisAGO2 or pFastbac-FlagMSI1 transformed DH10Bac cell, transfected into Sf9 insect cells to produce baculovirus. For isolating recombinant proteins, High Five insect cells were infected with gene-containing baculovirus for 48hr. The infected cells were harvested and washed in ice-cold PBS, lysed in Lysis buffer (20 mM Tris-HCl pH 7.9, 0.5 mM EDTA, 300 mM KCl, 10% Glycerol, 0.2% TritonX100, 10μM MG132)at 4℃ for 30 min. Crude lysate were centrifuged at 13K rpm (20000 xg) and recombinant proteins were isolated by Nickel (Quiagen) or anti-FLAG M2 (Sigma) resins and eluted in Lysis buffer (with 100 mM KCl) containing 100 mM imidazole or 150 ug/mL 3X FLAG peptide, respectively. For pull-down assay, 2 ug HisAGO2 and 2 ug FLAG-MSI1 were incubated as indicated in lysis buffer (with 100 mM KCl) at 4℃ for 2 h before pull-down by Protein A-immobilized anti-AGO2 antibody. After extesive wash with lysis buffer (100 mM KCl), the precipitated proteins were separated by gel electrophoresis and analyzed by immonoblotting with indicated antibodies.

RNA-binding protein immunoprecipitation (RIP)

Magna RIP kits (Millipore, Merck Co., Berlin, Germany, Catalog No. 17-700) [5] was used for RNA-binding protein immunoprecipitation and RNA extraction. The 05MG cells were washed twice with ice-cold PBS and cells were collected with 10 ml PBS by cell scraper. Collected cells were pelleted with 10-min centrifugation by 1500 rpm at 4℃. The pellets

were then resuspended in an equal volume of RIP lysis buffer (RIP lysis buffer (CS203176), protease inhibitor Cocktail (CS203220) and RNase Inhibitor (CS203219). The magnetic beads were prepared with 2.5 μl antibody for 30 minutes at room temperature, and the protein lysates were mixed with beads-antibody complex in 900 μl of RIP immunoprecipitation buffer (35 μl of 0.5M EDTA (CS203175), 5 μl RNase inhibitor and 860 μl RIP wash buffer (CS203177)) overnight with rotating at 4℃. The beads were washed thrice by ice-cold RIP wash buffer prior to the RNA isolation, followed by the RNA purification by performing proteinase K digestion at 55℃ for 30 mins with vigorous shaking. The supernatant was placed into a new tube and add 250 μl RIP wash buffer. Equal volume of phenol: chloroform: isoamyl alcohol (25:24:1) was added for RNA isolation. Vortex for 15 seconds and centrifuge at 14000 rpm for 10 mins to separate the phases. Move 350 μl of the aqueous phase into new tube and add 400 μl of chloroform. Vortex for 15 seconds and centrifuge at 14000 rpm for 10 mins. Remove 300 μl the aqueous phase into new tube and add 50 μl Salt Solution I (CS203173), 15 μl Salt Solution II (CS203185), 5 μl of Precipitate Enhancer (CS203208) and 850 μl absolute ethanol and freeze the samples at -80℃ overnight. At the very next day, each sample was centrifuged at 14000 rpm for 30 mins, removed supernatant, and washed pellets with 80% ethanol and centrifuge at 14000 rpm for 15 mins. Removed supernatant and air dry the pellets. The isolated RNAs were then resuspended in 20 μl of RNase-free waster (CS203217). All the antibodies and PCR primers used in this section were listed in Table S 7 [6].

Modified RNA-binding protein immunoprecipitation (modified-RIP)

We used RNA ChIP-IT kit (Catalog No. 53024) [7] for a modified-RIP assay to study the interaction regions of RNA-binding proteins on their target RNAs. Add 175 μl of 37% formaldehyde per 6.5 ml medium of sample in culture dish (final concentration has to be

approximately 1%) for 5 mins to fix the samples. Then add 825 μl Glycine to the sample (final concentration has to be 0.125M) for 5mins at room temperature to stop fixation. Remove the supernatant and discard. Washed the cell pellet and collected by centrifugation at 1000rpm for 5 mins at 4°C. Resuspend cells in ice-cold Complete Lysis Buffer, incubate on ice for 30 mins and transfer the cell by centrifugation at 5000rpm for 10 mins at 4°C. Remove the supernatant and resuspend the pellet in complete shearing buffer. Submit the samples to sonication to shear the chromatin using the Bioruptor® for 1 to 4 run of 5 cycles: [30 seconds "ON", 30 seconds "OFF"] each (20 cycles). Spin the control and sonicated samples at 12,000 rpm for 10 minutes. The supernatant, except the upper lipid layer, is collected. Treat the chromatin with 10μl DNase I for 20 mins at 37°C and stop the reaction by adding 10 10μl 0.5M EDTA before performing the IP.

First, the Dynabeads Protein-G was incubated with 2.5μl antibody 30 minutes at 4°C. Next, the protein lysis 1mg incubated with protein-G-conjugated-antibody beads overnight at 4°C for parental cells. Dynabeads Protein-G was separated by Complete RNA-ChIP Elution Buffer by rotate for 15 mins in the end-to-end rotor at room temperature. Transfer the supernatants and add 2μl 5M NaCl and 2μl proteinase K to each sample for 1h at 42°C to digest the proteins. Then, incubate for 1.5 hrs at 65°C to reverse the cross-links. RNA was extracted with phenol/chloroform/isoamyl alcohol, dissolved in 20 μl of KAPA distilled water, and used as a source of RNA for End point RT-PCR analysis (KAPA SYBR FAST Universal One-step qRT-PCR kit, KR0393). Each experiment was done in three distinct biological replicates. Quantification of fold changes of the signals was done by normalizing to IgG-precipitated controls. All the antibody and PCR primer were followed Table S 7 and 8.

RNA-Fluorescence in situ hybridization (RNA-FISH)

The cells were sub-cultured on 18 mm around coverglass in a 12-well cell culture plate 24h. After overnight cultured, cells were stimulated hypoxia. The cells were fixed with 3.7 %

formaldehyde for 5 mins. Permeabilized with 0.1% Triton X-100 for 5 mins at room temperature. The immune-stained with the indicated primary antibodies in hybridization buffer (Biosearch Technologies Cat#SMF-HB1-10) overnight at 4°C, respectively, followed by FITC-labeled or PE-labeled secondary antibodies in wash buffer A (Biosearch Technologies Cat#SMF-WA1-60). Finally, DAPI nuclear stain (wash Buffer A of 5ng/mL DAPI) to counterstain the nuclei that allowed imaging. The antibody used in this study was listed in Table S 7 and the RNA FISH probes as below: Human TP53 with Quasar 670 Dye (Cat.VSMF-2423-5) and Human CCND1 with Quasar 670 Dye (Cat.VSMF-2047-5) [8].

Immunofluorescence (IF) staining

Cells were sub-cultured on glass coverslips or chamber slides 24 hrs prior to the experiment. Cell were then subjected to undergo hypoxia with designated time in complete culture medium. The cells were fixed with 4 % paraformaldehyde for 10 mins. Permeabilized with 0.1% Triton X-100 for 10 mins and incubated with blocking buffer (5% BSA) for 1 hour at room temperature. The immune-stained with the indicated primary antibodies overnight at 4°C, respectively, followed by FITC-labeled or PE-labeled secondary antibodies for imaging. The secondary antibodies used in this study were listed in Table S 8.

Fluorescence resonance energy transfer (FRET) assay

The plasmids who generate fluorescent fusion protein, MSI1-pmOrange and AGO2-EGFP [9], were co-transfected into GBM cells. Twenty-four hours after transfection, cells were stimulated with hypoxia. The cells were washed twice with ice-cold PBS and fixed using 4 % paraformaldehyde for 10 mins. Photo-bleaching was performed by 514-nm wavelength laser exposure at the maximal intensity. An excitation wavelength of 488 nm and an emission wavelength of 520 ± 20 nm were used for GFP, and an excitation wavelength of 555 nm and an emission wavelength of 580 ± 20 nm were used for mOrange spectrum. The

FRET energy transfer efficiency (E_f) was calculated as $FRET_{eff}=(I_{post}-I_{pre}) / I_{post}$ where I_{pre} and I_{post} are the total fluorescence of the ROI before and after bleaching [10].

Liquid chromatography–mass spectrometry (LC-MS/MS) analysis

LC-MS/MS analysis was performed through the application of LTQ Orbitrap (Thermo Fisher Scientific Inc., Waltham, MA, USA) as previously described. In brief, each sample of digested peptides was reconstituted to 20 μl of 0.1% formic acid (FA). Peptides were firstly injected in and separated by the nanoflow HPLC (Agilent 1100, Agilent Technologies, Santa Clara, CA, USA) with a C18 column (75 μm ID × 360 μm OD × 15 cm; Agilent Technologies, Santa Clara, CA, USA), and became ionized particles once passed through the succeeding nanospray tip (New Objective, Woburn, MA). In operating HPLC, the flow rate was at 0.4 μl/min after a splitter. LC gradient for the LC-MS/MS system ramped from 2% ACN to 40% ACN in 120 min, and the system was performed under the setting of automated data-dependent acquisition, with mode of 200-2000 m/z full scan for the maximum 3 most intense peaks from each Orbitrap MS scan. Peptides with +2 or +3 charge state were further subjected to CID. Spectra were obtained in raw data files with Xcalibur (version 2.0 SR2). Protein identification was accomplished via TurboSEQUEST (Thermo Finnigan, San Jose, CA, USA) using the UniProt database. A protein was confirmed once 3 peptides with Xcorr > 2.5 were matched in sequencing [11]. The peptide sequences identified by Mass Spectrometry of the top 5 stress response-related proteins (Suppl. Figure 4b) are listed in Table S 1.

Split luciferase reconstitution reporter assay

To use gaussia luciferase (Gluc) for detecting protein-protein interaction, we split gaussia luciferase into NGluc (N-terminal Gluc, 106 a.a.) and CGluc (C-terminal Gluc, 79 a.a.) [12, 13]. The two fragments were amplified by polymerase chain reaction (PCR) and subjected

to construct fusion protein with MSI1 and AGO2 by the pcDNA 3.1 and pCMV backbone, respectively. Each fusion protein contains a flexible linker $(GGGGS)_2$ between the protein and polypeptides of split luciferase [14, 15]. Stable cell lines were obtained by stable transfection of both fusion protein expressive plasmids in 05MG GBM cell line with Hygromycin B (Sigma Aldrich Co., St. Louis, MI, USA) and G418 sulfate (Merck Co., Berlin, Germany). To establish a normalizing standard, we transduced multiple reporter genes into the aforementioned stable cell line for stably expressing green fluorescent protein (GFP), firefly luciferase (FLuc) and herpes simplex virus type I thymidine kinase (HSV1-tk) using lentivirus as previously described [16]. For in vitro study, the cells were lysed in mild reporter lysis buffer (Promega Co., Madison, WI, USA) with a frozen-thaw cycle. The supernatant was collected after brief centrifugation and dispensed in 96-well black flat bottom plate. Coelenterazine (Nanolight Technologies, Ltd., Pinetop, AZ, USA), the substrate of GLuc, was firstly dissolved in methanol and diluted in reporter assay buffer (15 mM potassium phosphate, 25 mM glycylglycine, 15 mM MgSO4, 4 mM EDTA). D-luciferin sodium salt (Promega Co., Madison, WI, USA) was dissolved in sterilized water and diluted in reporter assay buffer supplemented with 2mM ATP. The bioluminescent signals were acquired by Wallac 1420 Victor^2 Microplate Reader (Perkin Elmer, Waltham, MA, USA) equipped with auto-dispenser to avoid rapid decay of GLuc. For in vivo study, the xenografted mice were anesthetized by isoflurane inhalation (1% in O_2 supplement) prior to the In Vivo Imaging System (IVIS 50, PerkinElmer, Waltham, MA, USA) acquisition. The coelenterazine was injected through tail vein for a total of 15 μg per mouse, and the image was acquired within 5 minutes. The mice would be recovered from luminescent status for 30-minute rest; after that, the mice would undergo an intraperitoneal injection of D-luciferin (150 mg/kg) for tumor size normalization. The region of interests (ROI) was automatically chosen by the Living Image 4.2 software and quantified as photon flux in a certain area per second (photons/s/cm^2).

Immunohistochemistry staining and immunoblotting (IHC)

Tumor specimens from mice were fixed with 4% paraformaldehyde (Sigma Aldrich Co., St. Louis, MO, USA). Section were deparaffinized and rehydrated before staining. Tissue antigen were retrieved by boiling in 10 mmol/L (pH 6) citrate buffer (Sigma Aldrich Co., St. Louis, MO, USA) for 10 mins. Sections were cooling down in PBS for 10 mins before treating with 3% H_2O_2. Sample were blocked in 5mg/ml BSA (Sigma Aldrich Co., St. Louis, MO, USA) in PBS for 30 mins before hybridizing with 100 dilute primary antibodies Signals were amplified by the TSA Biotin System (PerkinElmer, Waltham, MA, USA) as instructed by the manufacturer and then counterstained with hematoxylin (Sigma Aldrich Co., St. Louis, MO, USA, #201708) for 30 mins [17]. The antibodies used in this study were listed in Table S 8.

Laser capture microdissection (LCM)

Serial sections (n = 3–20, 8 μm) were cut from each formalin-fixed paraffin-embedded (FFPE) sample and stored at 4°C until use. A 4-μm thick section was cut for H&E staining. Immediately before LCM, the sections were deparaffinized, stained with hematoxylin for 1 minute, dehydrated through alcohol gradients for 30 seconds each, and finally immersed in xylene for another 3 minutes and air-dried. The microdissection was performed using ArcturusXT Laser Capture Microdissector (Applied Biosystems-Life Technologies, Carlsbad, CA, USA) following the manufacturer's instrument. AutoScan™ analysis software module was implemented when using the ArcturusXT LCM instrument, which allowed the user to visually inspect the regions of interest. Approximately 5000 cells were captured per specimen and subsequently used for the following studies. Two 5-μm-thick sections were cut from each block and placed in sterile 1.5-mL centrifuge tubes for extraction. Tubes containing cut FFPE sections for RNA purification were stored at −80°C until use. Total RNA including small RNAs was extracted using FFPE RNA Isolation Kit (Life Technologies

Corporation, Carlsbad, CA, USA) following the instruction. RNA yield was determined from the A 260/A 280 absorbance ratios using a NanoDrop ND-1000 spectrophotometer (Thermo Fisher Scientific, Waltham, MA, USA).

Statistical analysis

Data are expressed as the mean ± SD from at least three independent experiments. The statistical analysis was performed using student's T-test. Difference were considered significant when $p \leq 0.05$ or $p \leq 0.01$.

Data Availability

Authors can confirm that all relevant data are included in the article and/or its supplementary information files.

SUPPLEMENTARY TABLES

Table S1. List of Peptide Sequences of the Top 5 Stress Response-related Proteins Identified from Mass Spectrometry

Gene Name	Protein Name	Peptide Sequence From Proteomic
PABPC1	Polyadenylate-binding protein 1 (PABP1)	DLFGK/QIYVGR/FGPALSVK/PASSQVPR /AVTEMNGR/SGVGNIFIK/AVNSATGVPTV/PLYVALAQR /GFGFVSFER/MNGMLLNDR/YQGVNLYVK/MNGMLLNDR /PAAAAAAATPAVR/FSPAGPILSIR/NLDDGIDDER/NFGEDMDDER /ALDTMNFDVIK/VANTSTQTMGPR/ALDTMNFDVIK/EFSPFGTITSAK /QAHLTNQYMQR/VDEAVAVLQAHQAK/GFGFVCFSSPEEATK /GYGFVHFETQEAAER/SLGYAYVNFQQPADAER
EIF2AK2	Interferon-induced, double-stranded RNA-activated protein kinase (PKR)	GVDYIHSK/DGIISDIFDK/IGDFGLVTSLK/DGIISDIFDKK /DLKPSNIFLVDTK/LTVNYEQCASGVHGPEGFHYK /RLTVNYEQCASGVHGPEGFHYK/AVSPLLLTTTNSSEGLSMGNYIG LINR
GCN	eIF-2-alpha kinase activator GCN	GAAYGLAGLVK/ALADENEFVR/ASLLDPVPEVR/YLLDSCAPLLR
EIF3A	Eukaryotic translation initiation factor 3 subunit A	ELEIEER/LESLNIQR/IGLINDMVR/NICQQVNIK
EIF2C2	Protein argonaute-2 (AGO2)	YCATVR/TPVYAEVK/SGNIPAGTTVDTK/NLYTAMPLPIGR

Table S2. Clinical manifestation and background of 18 GBM patients with primary and recurrent tumors.

	**Recurrent GBM**
No. of patients	18
Age (years)	62.1±5.7
Female	16
Survival (months)	4.1±0.2
KPS	
≥80	0
<80	18
P53 mutation	14
MGMT methylation	11
Surgery	
Total gross removal	18
Subtotal removal	0
No surgery	0
Radiation	18
Treatment with Temodal®	18

Table S3. Clinical manifestation and clinical background of a cohort of 67 primary and 32 recurrent GBM patients.

	GBM	Recurrent GBM
No. of patients	67	32
Age (years)	60.2±6.5	64.6±5.1
Female	30	18
Survival (months)	15.3±1.6	6.2±1.1
KPS		
≥80	29	0
<80	38	32
P53 mutation	60	25
MGMT methylation	41	19
Surgery		
Total gross removal	65	31
Subtotal removal	0	0
No surgery	2	1
Radiation	67	32
Temodal®	67	32

Table S4. Clinical manifestation and clinical background of a cohort of 61 recurrent PDAC patients.

	Non-recurrent	Recurrent
No. of patients	18	61
Age (years)	64.6±12.4	67.8±13
Sex		
Female	6	22
Male	12	39
Survival (months)		
DFS	62.87	10.3
OS	88.9	21
AJCC TNM status		
Stage 0	0	0
Stage IA	2	0
Stage IB	3	7
Stage IIA	8	14
Stage IIB	5	37
Stage III	0	3
Stage IV	0	0

Table S5. List of the primers used for plasmid construction

Name	Sequence (5'-3')
MSI-F	ATGGAGACTGACGCGCCCCAGCCCG
MSI1-R	TCAGTGGTACCCATTGGTGAAGGCT
MSI1-F-HindIII	AGAAGCTTATGGAGACTGACGCGCCCCAGC
MSI1-R-BamHI	AGGATCCTCAGTGGTACCCATTGGTGAAGG
MSI1-NLS-MutA-F	CGGGACCCCCTGACCGCAGCATCCGCAGGTTTCGGCTTCGTC
MSI1-NLS-MutA-R	GACGAAGCCGAAACCTGCGGATGCTGCGGTCAGGGGGTCCCG
MSI1-NLS-MutB-F	CCCAAGATGGTGACTGCAACGGCAGCAATCTTTGTGGGGGGGCTGTCG
MSI1-NLS-MutB-R	CGACAGCCCCCCCACAAAGATTGCTGCCGTTGCAGTCACCATCTTGGG
MSI1-NES-Mut-F	CCAGTCCTCCCCGAGGCAACAGCCGCACCTGCAACTGCCTACGGACCA
MSI1-NES-Mut-R	TGGTCCGTAGGCAGTTGCAGGTGCGGCTGTTGCCTCGGGGAGGACTGG
MSI1-C-term-F	ATCGAAGCTTTGCCCTACGGAATGGACGCC
MSI1-C-term-R	TAAGGGATCCTCAGTGGTACCCATTGGT
MSI1-F-Hind III	AATTAAGCTTATGGAGACTGACGCGCCCCAG
MSI1-R-BamHI-T1	TTAAGGATCCAACTGCTGACCCCCGAGTCC
MSI1-R-BamHI-T2	TTAAGGATCCATGGCTGTAAGCTCGGGG
MSI1-R-BamHI-T3	TTAAGGATCCAACTCCGGCTGGCGTAGG
MSI1-R-BamHI-T4	TTAGGATCCCCGTTGGCGACATCACCT

Table S6. Sequences of the primer used for real-time PCR analysis

Gene Name	Forward sequence	Reverse sequence
MSI1	TTG ACA AAA CCA CCA ACC GG	CCT CCT TTG GCT GAG CTT TCT T
p21	AGTACCCTCTCAGCTCCAGG	TGTCTGACTCCTTGTTCCGC
TP53	GGCAGGAAGGCTCCAGATG	CCTCACTGTTCATATGCCCATTC
CCND1	GAAGTTGCAAAGTCCTGGAGC	TGGTTTCCACTTCGCAGCA
CDK4	TCGTCGAAAGCCTCTCTTCTG	AGGCAGAGATTCGCTTGTGT
BIRC5	GAATTCCGGGACCCGTTGG	CCAAGTCTGGCTCGTTCTCA
AkT1	GCAGCACGTGTACGAGAAGA	CCTCCAAGCTATCGTCCAGC
MBP	CCAGGATTTGGCTACGGAGG	TAGGTAACAGGGGCAAGTGG
TMBIM6	AGGCGGGTTAGGAAGAGTGG	GACCATATGGACATAGGCCCC
cdc20	TGGGTTCCTCTGCAGACATTC	GCTCCTTGTAATGGGGAGACC
cdc6	CAGTTCAATTCTGTGCCCGC	GCTCCTTCTTGGCTCAAGGT
Hells	TTCCCGGGTGAGTGTCCAG	TATCCCAAGACATGCGAGCC
DLGAP5	TTCTTGCTGGTGGAGTAGCAG	TAGACCTGGTGAATCAAGAAGG
DCTN1	ACTGAAGCCTAAGAAGGCACC	CTCCAGGAGAGGTGAGGACC
β-actin	GCGTGACATTAAGGAGAAG	GAAGGAAGGCTGGAAGAG
GAPDH	AGAAGGCTGGGGCTCATTTG	AGGGGCCATCCACAGTCTTC
18S rRNA	CAGCCACCCGAGATTGAGCA	TAGTAGCGACGGGCGGTGTG
Septin 11	CTAATAAAGCGGGAGGGGCG	GTCCTATTTCAGTAGTACAGACGC
Septin 2	GTGGTGGGCTAGACGAGTTTC	CTTGCGGTGGGTAACTGGAG
ACTN4	GGCACAGACCAGAGCTGATT	TCCAGCATCTTGGGGATGTC
ALDH1A3	TCGACCTGGAGGGCTGTATTA	CAGGACCATGGTGTTCCCAC
ANXA5	ACCTGCCTACCTTGCAGAGA	CTTCCCCGTGACACGTTAGT
ARHGEF12	AAAGGAGGACCTCTCGCCAA	GCTGAACAAGACCTGTGGGG
BCAT1	TGATGCAATCCGCTAGGTCG	GCATCCGTTACTGCAATCCTTC
BCL2L1	GAAACGACCTGGCCGATGAA	GCTCCCGGTTACTCTGAGAC
CALM1	CAGTGGTGCTGGGAGTGTC	GATCAGCCATGGTGCGAGC
CALM2	AGGGAGGTGTTTATGAGGCG	ACAAAGCTAACCATGCTGCAA
CALM3	GATGCAGATGGGAACGGGA	AAAGACACGGAACGCCTCTC
CFL1	CTCATTGTGCGGCTCCTACTAA	AGAAGAGCACCGCCTTCTTG
CUL4B	GCAGAATCAGAATGTTCCGGG	CCTGGAGTTCCTTTTTACCCTCT
DAPK3	AATCTGAGGAGCTGGGTTGC	TGATGAACTTGGCTGCGTACT
DCTN2	TCGATGCGTTTGCACAAGA	CTGGGGTGTCTCCTTCACTC

DNAJC5	CCTATCGGAAGCTTGCCTTGA	CAGCTGGACAGCACGAAGTA
EID1	CTGGATGGCGGGTTTCAGAT	AGTTGGGTCCCTCCTCAAGT
EIF4G2	CCATTCGGGGAGACTCTGGT	ACCTCCATAGAGCTCCGACT
EIF5A	GCTCGGGTCCTAATCACCCC	TGCATCTCCTGTCTCGAAGTC
GPX1	TTTGGGCATCAGGAGAACGC	CAACATCGTTGCGACACACC
GSTP1	AGACCAGATCTCCTTCGCTGA	TCACTGTTTCCCGTTGCCAT
IRAK1	GAGTGGCTTTGAGAAGCACC	TCTAGCCTCTCGTACACCTGG
LGALS1	CTGGAAGTGTTGCAGAGGTGT	CCGTCAGCTGCCATGTAGTT
MACF1	GATCTTACAGGAGCGAGCGG	TGTGCTTGCGGACCTTCATT
MAPRE1	TTCTGCCGAGAGCCGAAGA	TTCAAGGCAATGGAGCCAGG
MCL1	TTCCAGTAAGGAGTCGGGGT	CCTCCTTCTCCGTAGCCAAA
MDM2	CAGCAGGAATCATCGGACTCA	TGTGGCGTTTTCTTTGTCGT
MFN2	GAAGGTGAAGCGCAATGTCCC	GTTCTTCTGTGGTAACGGGGT
NACC1	CTTCTTTGACCGGAACACGC	AGTACTTGACAGCGTGGAGC
NME1-NME2	AAGGAACCATGGCCAACTGT	AGATCTTCGGAAGCTTGCAT
NPM1	ACTCCAGCCAAAAATGCACA	CATGTAGTGCCCAGGACTGT
NPM1	CGGTTGTGAACTAAAGGCCG	TTTGCACCAGCCCCTAAACT
PAFAH1B1	ACGAGATGAACTAAATCGAGCTAT	TGACCAAGAGGTCCACCTGA
PPP1CB	AGCTCATCAGGTGGTGGAAGA	CGGAGGATTAGCTGTTCGAGG
PRC1	ACCTATTCTGAGTTTGCGAAGGA	TGATCAGGGCTTCTCAGGACT
PRDX1	CCCCACGGAGATCATTGCTT	AAAGGCCCCTGAACGAGATG
PSMB7	TTCCCAGAGTTGTGACAGCC	GCCAGAACCCATGGTGACAT
PSMD2	CGCGAGTTGGTCTGGGAAAA	CCTCTTCAGACAGCTCCTGTTC
RCC2	AAGTGTATCTGGTGAGTGGGC	GGAGTGATGAGAAACCGGAGA
RHOA	CGTTAGTCCACGGTCTGGTC	ACCAGTTTCTTCCGGATGGC
RNA	TTCTGGAAGGAACGCCGC	TGGTGTGGAACACTAGGGGA
RPL11	GAAGGGTCTAAAGGTGCGGG	ATGCTGAAACCTGGCCTACC
RPS3	GCGAGTTACACCAACCAGGA	CCCTCTGGAAAGCCAAACCT
RPS6	AAGCACCCAAGATTCAGCGT	TAGCCTCCTTCATTCTCTTGGC
RRM2B	GTAGCTTCGGCGGAGTCTG	AGTCGACCTCTTCTGCTGTC
S100A6	CGACCGCTATAAGGCCAGTC	GCAGCTTCGAGCCAATGGT
SOD1	ACAAAGATGGTGTGGCCGAT	AACGACTTCCAGCGTTTCCT
SPIN1	GGGTGGAAAGAGGGGAATGG	TGTGCATCGCTGATTCGAGA
SQSTM1	CCGTGAAGGCCTACCTTCTG	TCCTCGTCACTGGAAAAGGC
STMN1	CCATTGTCTGAAGGGACGGG	GACAAGCGACAGGCAGTGTA

TGM2	AGTCCCTGGAAATGCCAGCC	TGTCTACACTGGCCTCGTAGT
TPT1	AGGGGCTGCAGAACAAATCA	AGACAGAAAGCGCAGGGATT
TUBB	GCGCTTATCGAAGTGTGGTC	TTCCCCTAGACACTCGCTCC
UBC	AGTAGTCCCTTCTCGGCGAT	GACGATCACAGCGATCCACA
UHMK1	ATTTCCGGCTTCTGGGACTC	CCATCGGTGTGGGTTAAGGG
USP22	CCCATCTTTGTCCGGCCTC	CCAGTTGTCCACCTTGAAGC
YWHAE	GGGTGACGGTGAAGAGCAGAA	TCAGTGACAATGGGGAGTTTCC
ZWINT	CTCCAGCTTCTGTATACCCTGC	AGTCAGAGGCCTTTTCTAGGAT

Table S7. Sequences of the primer used for modified-RIP assay.

	Forward sequence	Reverse sequence
TP53-3'UTR-1	CTGAACAAGTTGGCCTGCAC	GGGACAGCTTCCCTGGTTAG
TP53-3'UTR-2	GGCCCACTTCACCGTACTAA	AGGGAACAAGCACCCTCAAG
TP53-3'UTR-3	GGTCGGTGGGTTGGTAGTTT	AGTCTTGGTGGATCCAGATCAT
TP53-3'UTR-4	ACCCTGTCTGACAACCTCTTGG	AGGCAGAGATTCGCTTGTGT
TP53-3'UTR-5	ACCCTGTCTGACAACCTCTTGG	ATGAACCTGTGGTCCCAGCT
TP53-3'UTR -6	GCCACCATGGCCAGCCAACT	CACCCCTCAGACACACAGGT
TP53-CDS-1	TGAAGCTCCCAGAATGCCAG	GCTGCCCTGGTAGGTTTTCT
TP53-CDS-2	TGTGACTTGCACGTACTCCC	ACCATCGCTATCTGAGCAGC
TP53-CDS-3	GACATAGTGTGGTGGTGCCC	ACAAACACGCACCTCAAAGC
TP53-UTR-4	TTTGAGGTGCGTGTTTGTGC	CCCACGGATCTGAAGGGTGAA
TP53-UTR-5	TTCACCCTTCAGATCCGTGG	CAGTGGGGAACAAGAAGTGGA
NF2-3'UTR-1	AGAGCTCTAGCAGGTGACCC	CAGGTCAGAGAACTAGAACGCC
NF2-3'UTR-2	ATGGCGTTCTAGTTCTCTGACC	ATGATGGCACTGGCTTCTCA
NF2-3'UTR-3	GAACATTCATTCCCCCACCG	CGAGTGCCCTGTACCATCAG
NF2-3'UTR-4	TGGCTGGGGAGAGACTTTAG	CACACAGGAAGGAGCGTCTAT
NF2-3'UTR-5	CGCCCATAGACGCTCCTTC	CAAAGTGAGGCCTGGGTACAA
NF2-3'UTR-6	TTGTACCCAGGCCTCACTTTG	GCCCCAGACCAAGGAGTGAG
NF2-3'UTR-7	TTTTCTCCATGGCTGATGCTG	AGCAGCCCAACCCCATTAG
NF2-3'UTR-8	CTGACCTAATGGGGTTGGGCT	AGAGCCAGACCTCACTTTACAA
NF2-3'UTR-9	TCAGTCTTGAAGCCCATCCCT	CTTGGCACTTCCCAGACTTCA
NF2-3'UTR-10	CTGAAGTCTGGGAAGTGCCAA	TCCTGCTACTGGGGCTTGAG
NF2-3'UTR-11	TAGGGCCTGGGAGTTTGTCA	GATGAACGAAGCCATCTGTGC
NF2-3'UTR-12	CCCCCAACCTGTGTTGTCC	GCAGCTGGTTGTCAGTCTCTG
NF2-CDS-1	GACGCCGAGATGGAGTTCAA	TGAAAGGTGACTGGTTCTTCCT
NF2-CDS-2	CAGTGTTCACAAGCGGGGAT	CACACCGTACATCTCCAGGTC
NF2-CDS-3	TCCCGTGGAATGAAATCCGA	GCTGAACTTCCAAAGAATCGGC
NF2-UTR-4	TTGGCTGAAAAGGCCCAGAT	TGCTTCAGCTGATCTGCCTC
NF2-UTR-5	TGACATGAAGCGGCTTTCCA	ACCCCTGTCGGAGTTCTCAT
CCND1-3'UTR-1	GCGTCTCGGGAGAGGATTAG	GCCTAGAACCCCACTACAGC
CCND1-3'UTR-2	CCCACAGCTACTTGGTTTGTG	TTTCTTCTTGACTGGCACGC
CCND1-3'UTR-3	CTGCGTGCCAGTCAAGAAGA	ACCTTCCGGTGTGAAACATC

CCND1-3'UTR-4	GCAGAGGATGTTCATAAGGCCA	GATGACTCTGGGAAACGCCA
CCND1-CDS-1	CTGCGAAGTGGAAACCATCC	AAGACCTCCTCCTCGCACTT
CCND1- CDS-2	GCCATGAACTACCTGGACCG	CAATGAAATCGTGCGGGGTC
CCND1- CDS-3	ACACTTCCTCTCCAAAATGCCA	TGTGAGGCGGTAGTAGGACAG
CCND1- CDS-4	GTGATCAAGTGTGACCCGGA	GCCCTCAGATGTCCACGTCC
HELLS-3'UTR-1	AAGTGGAGCTCAAGAATAGCTT	TCTTTGTTCTTGGTAAGGCTCAGA
HELLS-3'UTR-2	ACTGATTGTCCACTTCACCTTTTT	AGTACACATCAGCCTGTATCCAA
HELLS-3'UTR-3	TCTTGGATACAGGCTGATGTGT	TCTCTCCCCATGAAAAGCCT
HELLS-3'UTR-4	AGTGATTTCCCTGTATTGGGTTT	TCTTTGTTCTTGGTAAGGCTCA
HELLS-3'UTR-5	ACAGGCTGATGTGTACTTAACCA	GCATAATCCCAATCTCTCCCCA
HELLS-CDS-1	CAGCGGCGGCTCGGA	CAGGTCAGAGAACTAGAACGCC
HELLS-CDS-2	TCGGTACCGTAGACTTCAACA	TGTTGCTGTTCCATTTTCGTCA
HELLS-CDS-3	TCAGAGGTCATGTCAAAAGAGGA	TCCTCTTTTGACATGACCTCTGA
HELLS-CDS-4	GACCCAGTCCGGAAGTGTAA	TACACTTCCGGACTGGGTCA
HELLS-CDS-5	TTGTCTGTGGCCCTTTGTCT	TGTAGACAAAGGGCCACAGAC
HELLS-CDS-6	CTTTTGACTGGTACTCCCTTGC	TCTGCAAAGTCCCTTTCCGT
HELLS-CDS-7	TCCACTTTCAAAGAAGCAGGAGA	GGAACTTCAAGAGCAACATCAGA
HELLS-CDS-8	CCGAGAAAGAGCTGTTGTGGA	ACAGCTCTTTCTCGGTCCAC
HELLS-CDS-9	AGGCTTGATGGGTCCATGTCT	AAAAAGCAGCACCTTGTGACC
HELLS-CDS-10	ACCCCCAGTCGGATCTTCAG	ATCCGACTGGGGGTTCCAA
HELLS-CDS-11	TTCAAAGGTGGTCAGTCTGGATT	TCCCCATCTTCTCTTTAATTGGT
HELLS-CDS-12	GGACCAATTAAAGAGAAGATGGGG	TGTTCTTGGTAAGGCTCAGAAA

Table S8. Antibody list

ANTIBODIES	SOURCE	IDENTIFIER
Rabbit monoclonal anti-Argonaute 2	Cell Signaling Technology	Cat#2897;
Mouse monoclonal anti-Argonaute 2	abcam	Cat#ab57113
Rabbit polyclonal anti-Argonaute 2	abcam	Cat#ab32381
Mouse monoclonal anti-β-Actin	SIGMA	Cat#a5316
Mouse monoclonal anti-Cyclin D1	abcam	Cat#ab6125
Rabbit monoclonal anti-CDK4	Cell Signaling Technology	Cat#12790
Rabbit polyclonal anti-HELLS	Cell Signaling Technology	Cat#7998
Rabbit polyclonal anti-HIF-1α	Cell Signaling Technology	Cat#3716
Rabbit polyclonal anti-Lamin A/C	Cell Signaling Technology	Cat#2032
Rabbit monoclonal anti-Musashi-1	Cell Signaling Technology	Cat#5663
Rabbit monoclonal anti-Musashi-1	abcam	Cat#ab52865
Rabbit monoclonal anti-Merlin	Cell Signaling Technology	Cat#12888
Rabbit monoclonal anti-p53	Cell Signaling Technology	Cat#2527
Rabbit monoclonal anti- p21 Waf1/Cip1	Cell Signaling Technology	Cat#2947
Mouse monoclonal anti-Flag M2	SIGMA	Cat#F1804
Rabbit polyclonal anti-DDDDK tag	abcam	Cat#ab1162
Mouse polyclonal IgG	Millipore	Cat#12-371
Rabbit polyclonal IgG	Millipore	Cat#12-370
EasyBlot anti-mouse IgG	GeneTex	Cat#GTX225857-01
EasyBlot anti-rabbit IgG	GeneTex	Cat#GTX225856-01
Anti-mouse IgG, HRP-linked Antibody	Cell Signaling Technology	Cat#7076
Anti-rabbit IgG, HRP-linked Antibody	Cell Signaling Technology	Cat#7074
Goat anti-Mouse IgG (H+L) Secondary Antibody, Alexa Fluor 488 conjugate	Invitrogen	Cat#A-11001
Goat anti-Mouse IgG (H+L) Secondary Antibody, Alexa Fluor 555 conjugate	Invitrogen	Cat#A-21424
Goat anti-Rabbit IgG (H+L) Secondary Antibody, Alexa Fluor 488 conjugate	Invitrogen	Cat#A-11008
Goat anti-Rabbit IgG (H+L) Secondary Antibody, Alexa Fluor 568 conjugate	Invitrogen	Cat#A-11036
Human TP53 with Quasar 670 Dye (RNA Fish)	Biosearch Technologies	Cat#VSMF-2423-5

Human CCND1 with Quasar 670 Dye (RNA Fish)	Biosearch Technologies	Cat#VSMF-2047-5
Annexin V	BD Pharmingen™	Cat#556547
DAPI	SIGMA	Cat#D9542

REFERENCES

1. Chiou GY, Chien CS, Wang ML, Chen MT, Yang YP, Yu YL, et al. Epigenetic regulation of the miR142-3p/interleukin-6 circuit in glioblastoma. Molecular cell. 2013; 52: 693-706.

2. Kawahara H, Okada Y, Imai T, Iwanami A, Mischel PS, Okano H. Musashi1 cooperates in abnormal cell lineage protein 28 (Lin28)-mediated let-7 family microRNA biogenesis in early neural differentiation. J Biol Chem. 2011; 286: 16121-30.

3. Roberts A, Pachter L. Streaming fragment assignment for real-time analysis of sequencing experiments. Nat Methods. 2013; 10: 71-3.

4. Langmead B, Trapnell C, Pop M, Salzberg SL. Ultrafast and memory-efficient alignment of short DNA sequences to the human genome. Genome Biol. 2009; 10: R25.

5. Keene JD, Komisarow JM, Friedersdorf MB. RIP-Chip: the isolation and identification of mRNAs, microRNAs and protein components of ribonucleoprotein complexes from cell extracts. Nature protocols. 2006; 1: 302-7.

6. Dahm GM, Gubin MM, Magee JD, Techasintana P, Calaluce R, Atasoy U. Method for the isolation and identification of mRNAs, microRNAs and protein components of ribonucleoprotein complexes from cell extracts using RIP-Chip. Journal of visualized experiments : JoVE. 2012.

7. Selth LA, Gilbert C, Svejstrup JQ. RNA immunoprecipitation to determine RNA-protein associations in vivo. Cold Spring Harbor protocols. 2009; 2009: pdb prot5234.

8. Raj A, van den Bogaard P, Rifkin SA, van Oudenaarden A, Tyagi S. Imaging individual mRNA molecules using multiple singly labeled probes. Nature methods. 2008; 5: 877-9.

9. Shen J, Xia W, Khotskaya YB, Huo L, Nakanishi K, Lim SO, et al. EGFR modulates microRNA maturation in response to hypoxia through phosphorylation of AGO2. Nature. 2013; 497: 383-7.

10. Karpova T, McNally JG. Detecting protein-protein interactions with CFP-YFP FRET by acceptor photobleaching. Current protocols in cytometry / editorial board, J Paul Robinson, managing editor [et al]. 2006; Chapter 12: Unit12 7.

11. Jiang BH, Chen WY, Li HY, Chien Y, Chang WC, Hsieh PC, et al. CHD1L Regulated PARP1-Driven Pluripotency and Chromatin Remodeling During the Early-Stage Cell Reprogramming. Stem cells. 2015; 33: 2961-72.

12. Kato N, Jones J. The split luciferase complementation assay. Methods Mol Biol. 2010; 655: 359-76.

13. Paulmurugan R, Umezawa Y, Gambhir SS. Noninvasive imaging of protein-protein interactions in living subjects by using reporter protein complementation and reconstitution strategies. Proc Natl Acad Sci U S A. 2002; 99: 15608-13.

14. Trinh R, Gurbaxani B, Morrison SL, Seyfzadeh M. Optimization of codon pair use within the (GGGGS)3 linker sequence results in enhanced protein expression. Mol Immunol. 2004;

40: 717-22.

15. Newton DL, Xue Y, Olson KA, Fett JW, Rybak SM. Angiogenin single-chain immunofusions: influence of peptide linkers and spacers between fusion protein domains. Biochemistry. 1996; 35: 545-53.

16. Lin LT, Chiou SH, Lee TW, Liu RS, Hwang JJ, Chang CH, et al. A comparative study of primary and recurrent human glioblastoma multiforme using the small animal imaging and molecular expressive profiles. Molecular imaging and biology : MIB : the official publication of the Academy of Molecular Imaging. 2013; 15: 262-72.

17. Chien CS, Wang ML, Chu PY, Chang YL, Liu WH, Yu CC, et al. Lin28B/Let-7 Regulates Expression of Oct4 and Sox2 and Reprograms Oral Squamous Cell Carcinoma Cells to a Stem-like State. Cancer research. 2015; 75: 2553-65.

Figure S1

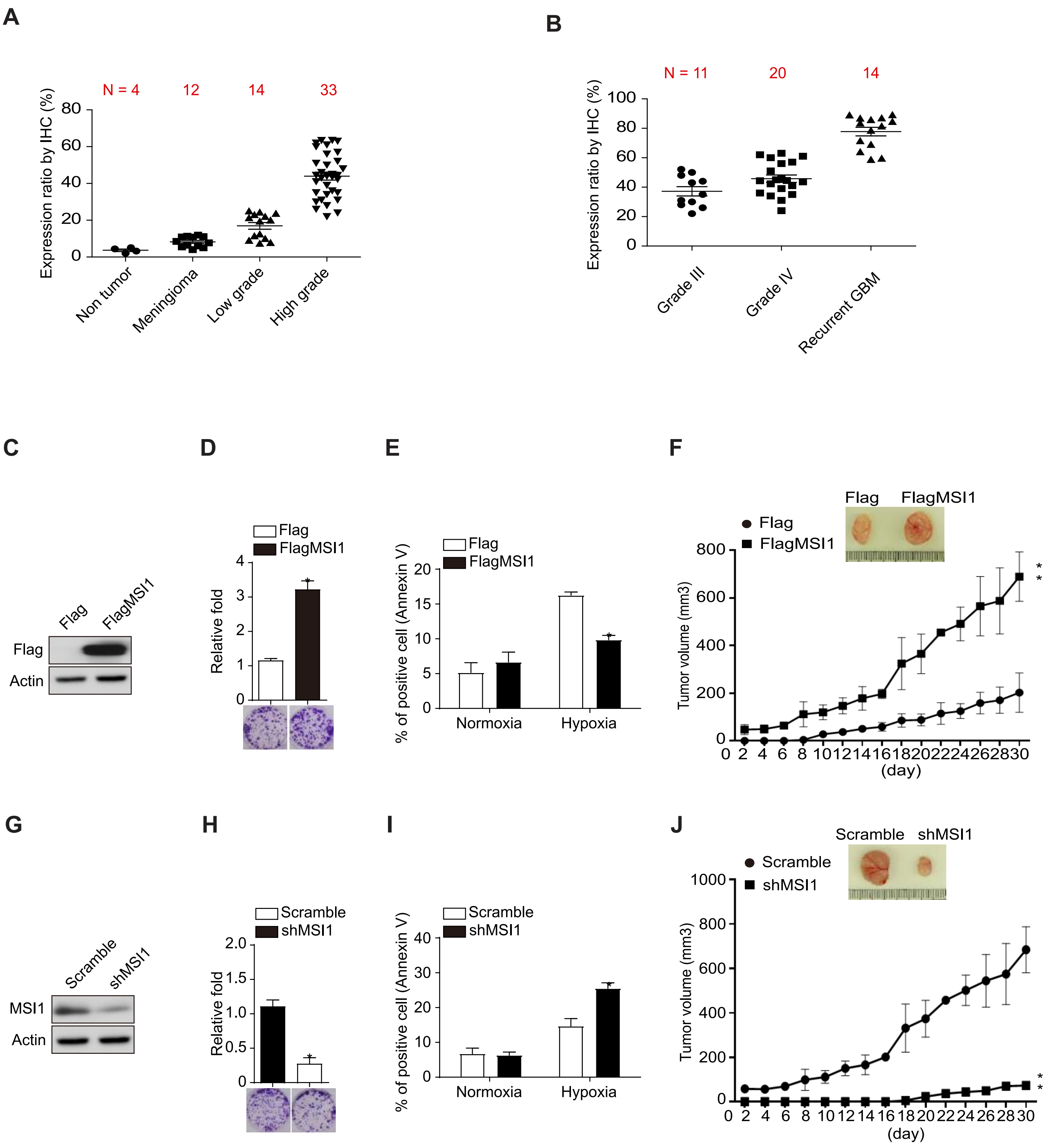
A
N = 4
12
14
33
Expression ratio by IHC (%)
Non tumor
Meningioma
Low grade
High grade
B
N = 11
20
14
Expression ratio by IHC (%)
Grade III
Grade IV
Recurrent GBM
C
Flag
FlagMSI1
Flag
Actin
D
Flag
FlagMSI1
Relative fold
E
Flag
FlagMSI1
% of positive cell (Annexin V)
Normoxia
Hypoxia
F
Flag
FlagMSI1
Flag
FlagMSI1
Tumor volume (mm3)
(day)
G
Scramble
shMSI1
MSI1
Actin
H
Scramble
shMSI1
Relative fold
I
Scramble
shMSI1
% of positive cell (Annexin V)
Normoxia
Hypoxia
J
Scramble
shMSI1
Scramble
shMSI1
Tumor volume (mm3)
(day)

Figure S2

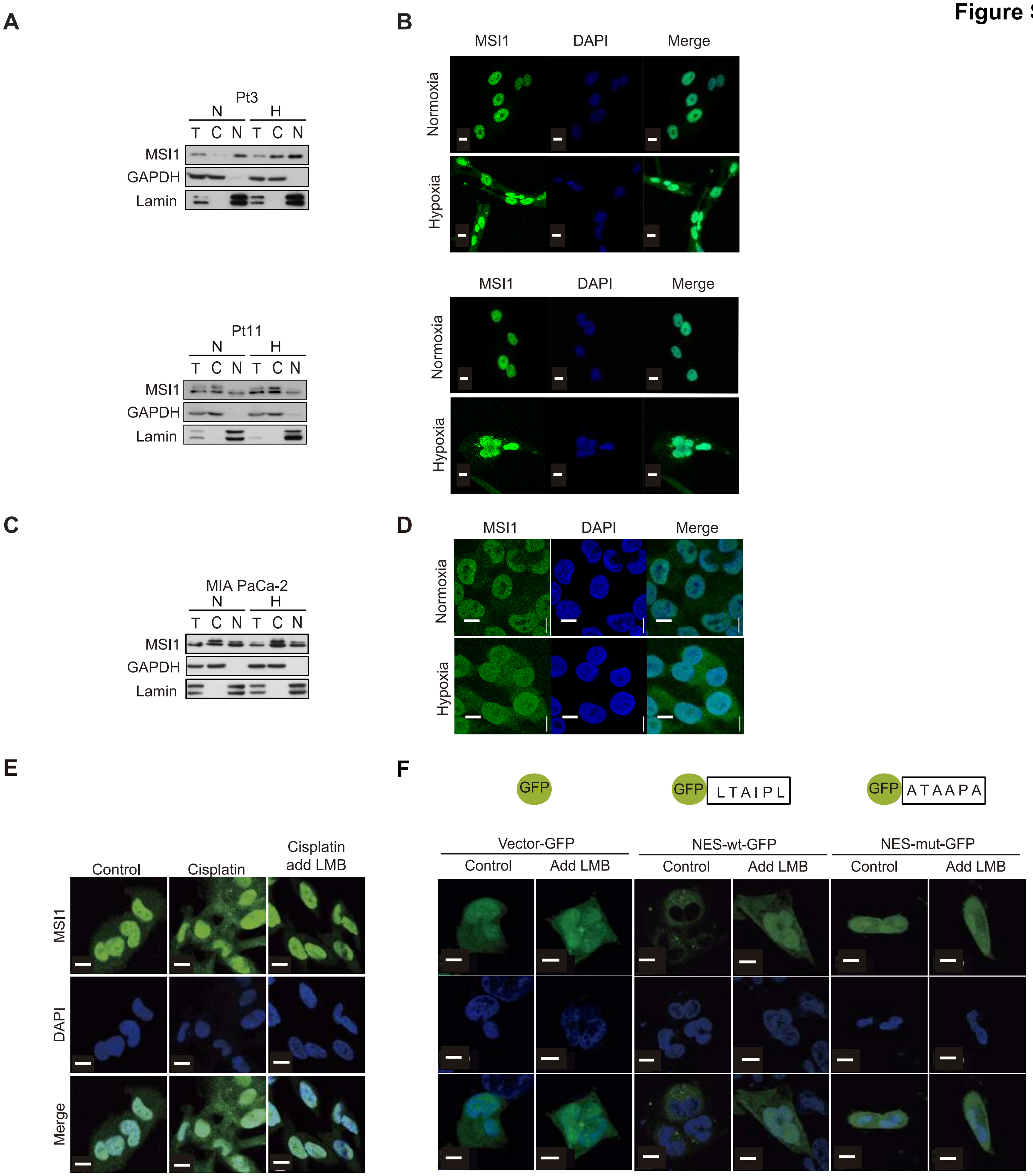
A
Pt3
N
H
T C N T C N
MSI1
GAPDH
Lamin
Pt11
N
H
T C N T C N
MSI1
GAPDH
Lamin
B
MSI1
DAPI
Merge
Normoxia
Hypoxia
MSI1
DAPI
Merge
Normoxia
Hypoxia
C
MIA PaCa-2
N
H
T C N T C N
MSI1
GAPDH
Lamin
D
MSI1
DAPI
Merge
Normoxia
Hypoxia
E
Control
Cisplatin
Cisplatin add LMB
MSI1
DAPI
Merge
F
GFP
GFP LTAIPL
GFP ATAAPA
Vector-GFP
NES-wt-GFP
NES-mut-GFP
Control
Add LMB
Control
Add LMB
Control
Add LMB

Figure S3

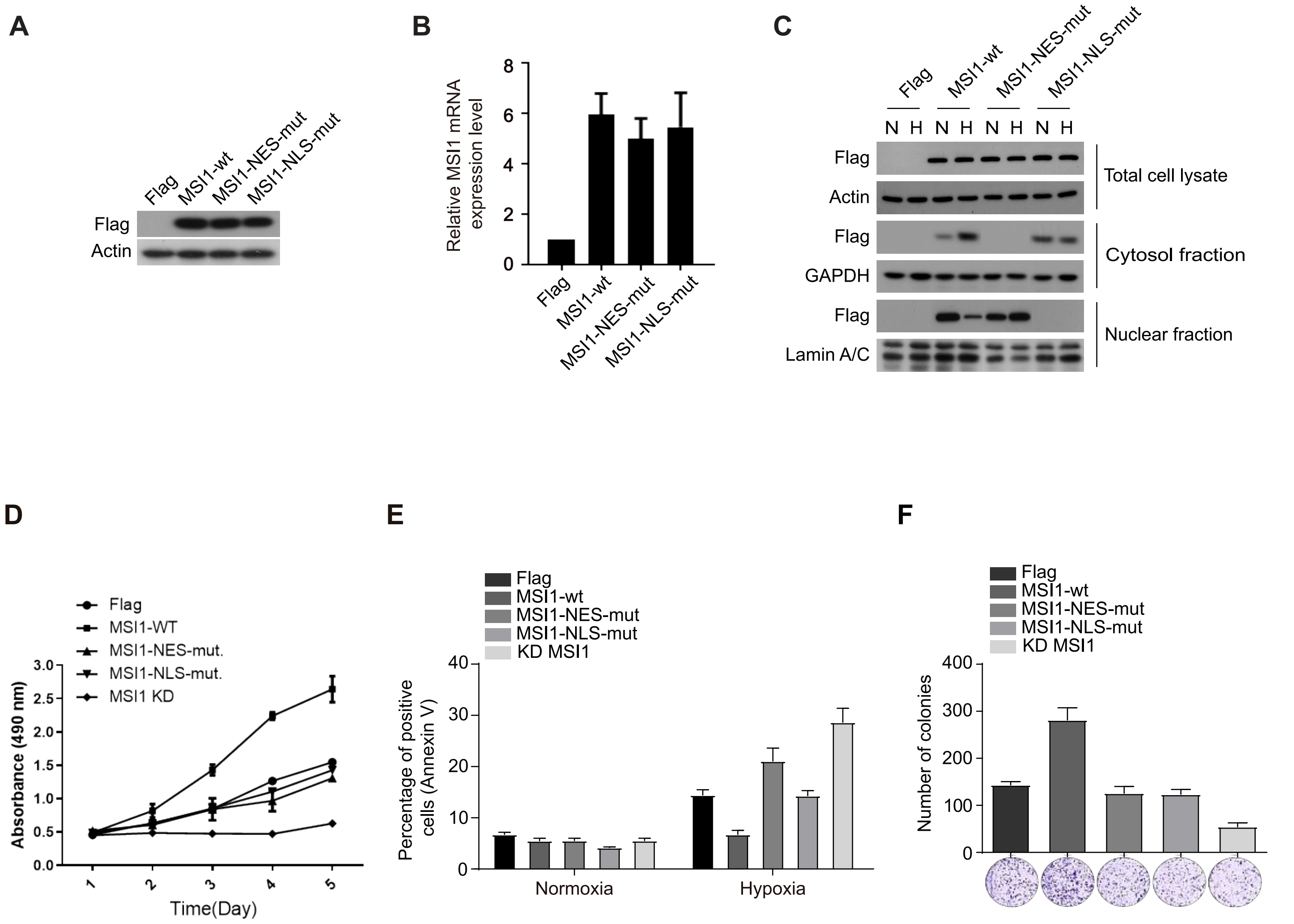
A
Flag
MSI1-wt
MSI1-NES-mut
MSI1-NLS-mut
Flag
Actin
B
Relative MSI1 mRNA expression level
8
6
4
2
0
Flag
MSI1-wt
MSI1-NES-mut
MSI1-NLS-mut
C
Flag
MSI1-wt
MSI1-NES-mut
MSI1-NLS-mut
N H N H N H N H
Flag
Actin
Total cell lysate
Flag
GAPDH
Cytosol fraction
Flag
Lamin A/C
Nuclear fraction
D
Flag
MSI1-WT
MSI1-NES-mut.
MSI1-NLS-mut.
MSI1 KD
Absorbance (490 nm)
3.0
2.5
2.0
1.5
1.0
0.5
0.0
1
2
3
4
5
Time(Day)
E
Flag
MSI1-wt
MSI1-NES-mut
MSI1-NLS-mut
KD MSI1
Percentage of positive cells (Annexin V)
40
30
20
10
0
Normoxia
Hypoxia
F
Flag
MSI1-wt
MSI1-NES-mut
MSI1-NLS-mut
KD MSI1
Number of colonies
400
300
200
100
0

Figure S4

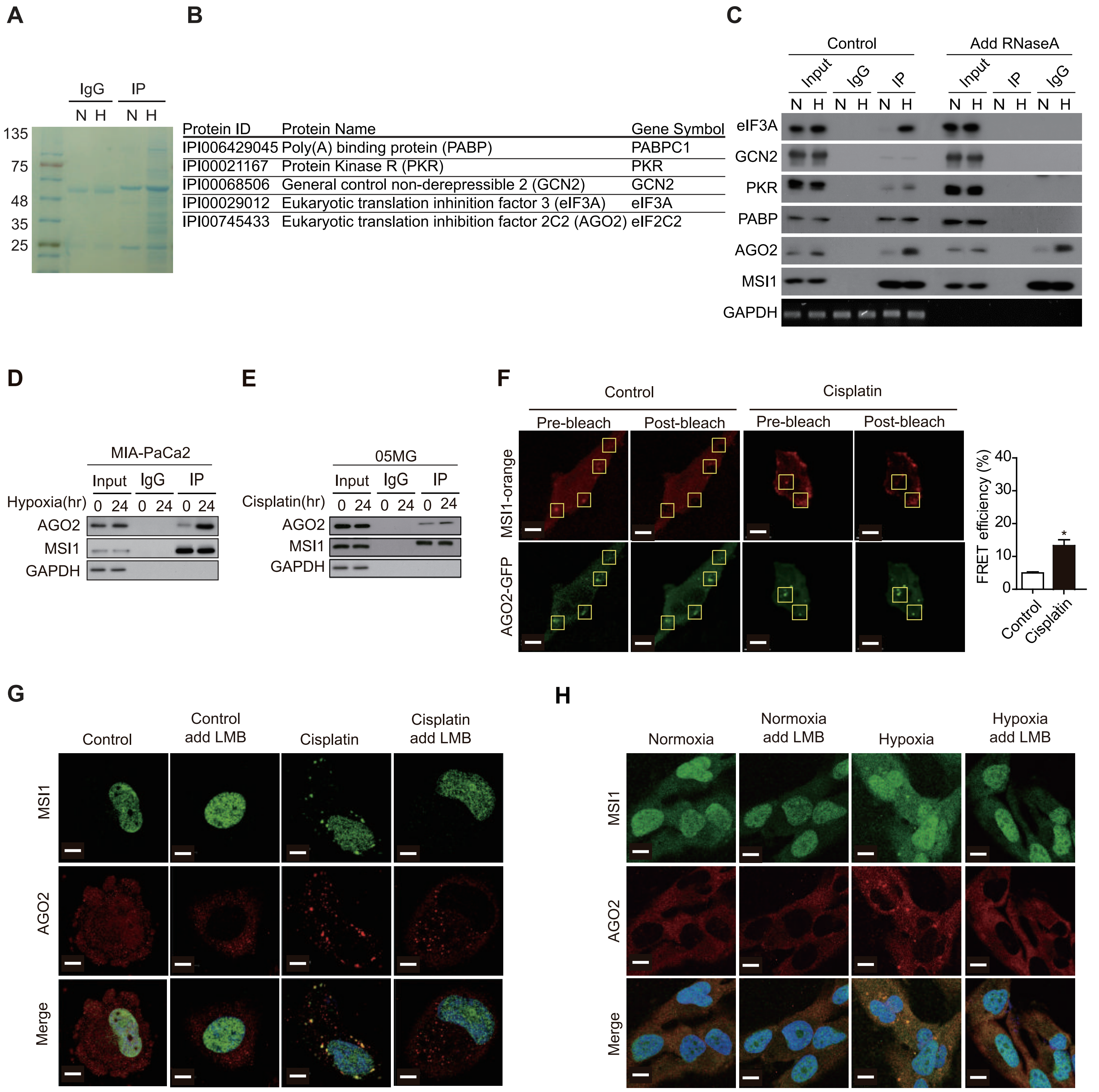

Protein ID	Protein Name	Gene Symbol
IPI006429045	Poly(A) binding protein (PABP)	PABPC1
IPI00021167	Protein Kinase R (PKR)	PKR
IPI00068506	General control non-derepressible 2 (GCN2)	GCN2
IPI00029012	Eukaryotic translation inhinition factor 3 (eIF3A)	eIF3A
IPI00745433	Eukaryotic translation inhibition factor 2C2 (AGO2)	eIF2C2

Figure S5

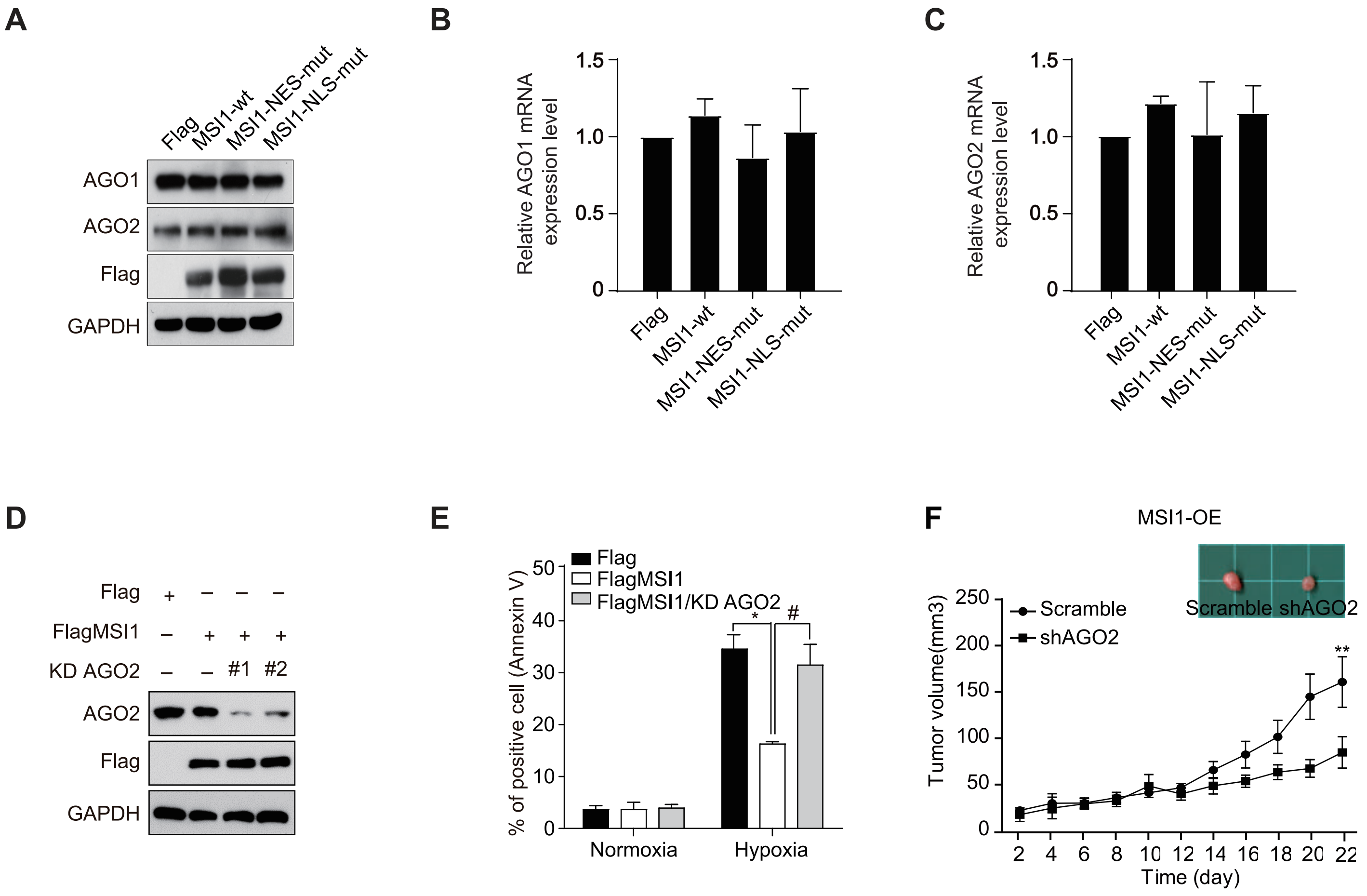

A
Flag
MSI1-wt
MSI1-NES-mut
MSI1-NLS-mut
AGO1
AGO2
Flag
GAPDH
B
Relative AGO1 mRNA expression level
C
Relative AGO2 mRNA expression level
D
Flag
FlagMSI1
KD AGO2
#1
#2
AGO2
Flag
GAPDH
E
Flag
FlagMSI1
FlagMSI1/KD AGO2
% of positive cell (Annexin V)
Normoxia
Hypoxia
F
MSI1-OE
Scramble
shAGO2
Tumor volume(mm3)
Time (day)

Figure S6

A

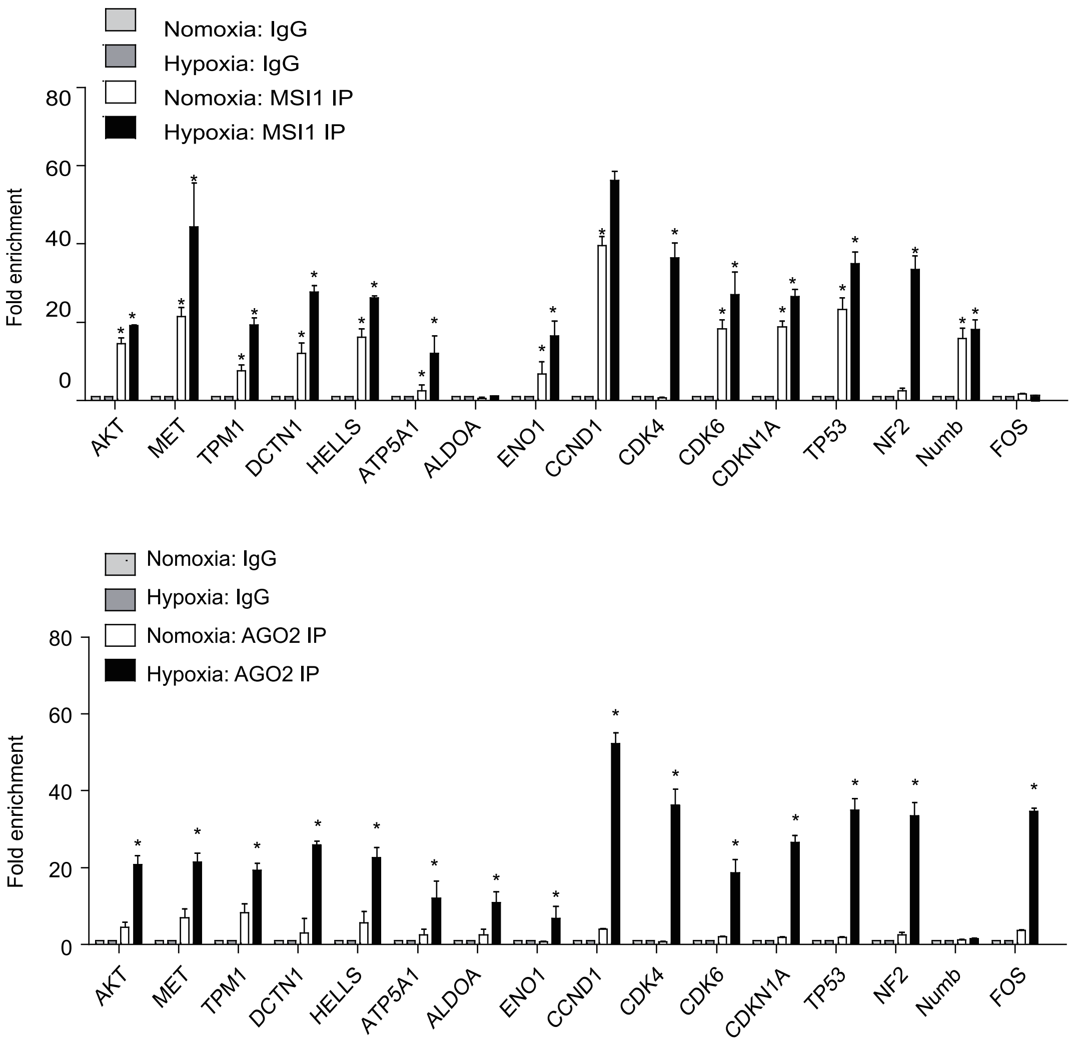
Nomoxia: IgG
Hypoxia: IgG
Nomoxia: MSI1 IP
Hypoxia: MSI1 IP
Fold enrichment
80
60
40
20
0
AKT
MET
TPM1
DCTN1
HELLS
ATP5A1
ALDOA
ENO1
CCND1
CDK4
CDK6
CDKN1A
TP53
NF2
Numb
FOS
Nomoxia: IgG
Hypoxia: IgG
Nomoxia: AGO2 IP
Hypoxia: AGO2 IP
Fold enrichment
80
60
40
20
0
AKT
MET
TPM1
DCTN1
HELLS
ATP5A1
ALDOA
ENO1
CCND1
CDK4
CDK6
CDKN1A
TP53
NF2
Numb
FOS

Figure S7

A

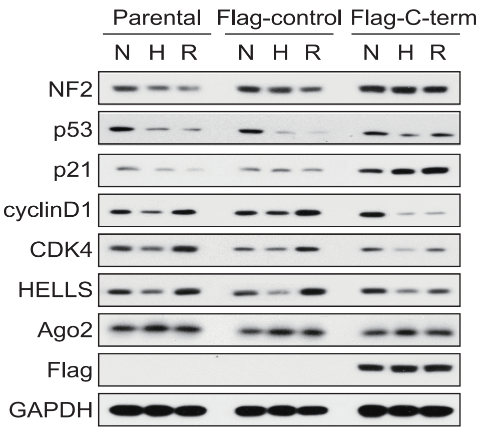
Parental
Flag-control
Flag-C-term
N H R N H R N H R
NF2
p53
p21
cyclinD1
CDK4
HELLS
Ago2
Flag
GAPDH

Figure S8

A

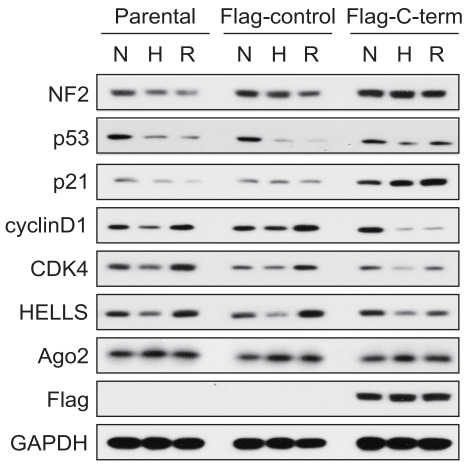

Parental
Flag-control
Flag-C-term
N H R N H R N H R
NF2
p53
p21
cyclinD1
CDK4
HELLS
Ago2
Flag
GAPDH
